# Indole is an essential herbivore-induced volatile priming signal in maize

**DOI:** 10.1038/ncomms7273

**Published:** 2015-02-16

**Authors:** Matthias Erb, Nathalie Veyrat, Christelle A. M. Robert, Hao Xu, Monika Frey, Jurriaan Ton, Ted C. J. Turlings

**Affiliations:** 1 Institute of Plant Sciences, Department of Biology, University of Bern, Altenbergrain 21, 3013 Bern, Switzerland; 2 Laboratory for Fundamental and Applied Research in Chemical Ecology, Faculty of Science, University of Neuchâtel, Rue Emile-Argand 11, 2009 Neuchâtel, Switzerland; 3 Lehrstuhl für Genetik, TU Munich, Emil-Ramann-Straβe 8, 85354 Freising, Germany; 4 Department of Animal and Plant Sciences, University of Sheffield, Western Bank, S10 2TN Sheffield, UK

## Abstract

Herbivore-induced volatile organic compounds prime non-attacked plant tissues to respond more strongly to subsequent attacks. However, the key volatiles that trigger this primed state remain largely unidentified. In maize, the release of the aromatic compound indole is herbivore-specific and occurs earlier than other induced responses. We therefore hypothesized that indole may be involved in airborne priming. Using indole-deficient mutants and synthetic indole dispensers, we show that herbivore-induced indole enhances the induction of defensive volatiles in neighbouring maize plants in a species-specific manner. Furthermore, the release of indole is essential for priming of mono- and homoterpenes in systemic leaves of attacked plants. Indole exposure markedly increases the herbivore-induced production of the stress hormones jasmonate-isoleucine conjugate and abscisic acid, which represents a likely mechanism for indole-dependent priming. These results demonstrate that indole functions as a rapid and potent aerial priming agent that prepares systemic tissues and neighbouring plants for incoming attacks.

## Abstract (web summary)

Herbivore attack of plant tissue primes non-attacked tissue to respond more strongly to subsequent attacks. Here, Erb *et al*. identify indole as an airborne priming signal that enhances herbivore-induced defensive-volatile and stress-hormone production in systemic tissues and neighbouring plants.

## Graphical abstract

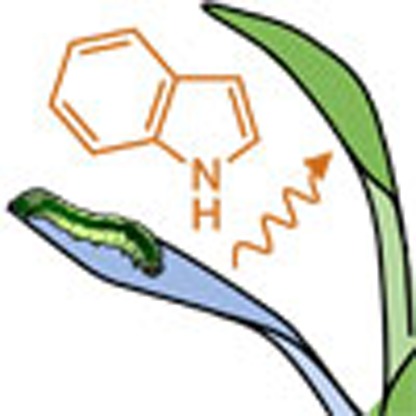

In response to herbivore attack, plants activate a wide array of defences that can reduce herbivore damage, including blends of volatile organic compounds (VOCs) that can be used as foraging cues by natural enemies of the herbivores^1^,^2^,^3^,^4^. Herbivore-induced plant volatiles (HIPVs) have also been implicated in plant–plant communication, as they can be perceived by neighbouring plants^5^,^6^, and prime them for an enhanced response upon subsequent insect attack^7^. By targeting jasmonic acid (JA)-inducible genes, HIPVs have been shown to enhance both direct and indirect defence responses^8^,^9^, which can benefit the receiver by decreasing herbivore damage^8^,^10^. However, the benefit for the emitter plant is not evident in this context, leading to the notion that plants do not communicate, but eavesdrop on each other^11^. As an adaptive explanation for why plants emit HIPVs, a role of HIPVs as within-plant signal has been proposed^12^. Indeed, HIPV-mediated within-plant communication has been demonstrated in several plant species including sagebrush, lima beans, poplar and blueberry^13^,^14^,^15^,^16^. In these cases, HIPVs released from an attacked part of the plant primed the healthy parts of the same plant to respond more strongly^15^,^17^. Within-plant communication through HIPVs is especially efficient when the vascular connectivity is limited or when adjacent leaves are spatially but not anatomically close^18^. As discussed by Heil and Ton^9^, herbivorous insects often move from one leaf to another, but adjacent leaves are not always directly connected via the plant’s vascular system. Therefore, volatile compounds may reach distal parts of the plant faster than vascular signals.

An important step to understand the mechanistic underpinnings of airborne and vascular systemic priming is the elucidation of the actual messengers^19^. Methylated forms of plant hormones, including methyl jasmonic acid and methyl salicylic acid, have been identified as volatile signals in this context^20^,^21^,^22^. In *Arabidopsis thaliana*, however, none of these signals are strictly required for systemic acquired resistance^23^ and the existence of other volatile priming agents has been proposed^24^. Other candidate volatiles that may prime systemic tissues are green leaf volatiles (GLVs) and terpenoids. Exposing lima bean (*Phaseolus lunatus*) leaves to volatiles from spider mite-infested lima bean leaves as well as to the terpenoids β-ocimene, (3E)-4,8-dimethyl-1,3,7-nonatriene (DMNT) or (3E,7E)-4,8,12-trimethyl-1,3,7,11-tridecatetraene (TMTT) resulted in the induction of defence-related genes^25^,^26^. However, in maize, there is no indication that terpenoids can prime defence responses in the receiver plants^27^. Evidence for GLVs as priming signals, on the other hand, has been found in multiple plant species, including maize. Exposure to (*Z*)-3-hexenyl acetate for instance was sufficient to induce extra floral nectar secretion in lima bean plants^28^. Treatment of *A. thaliana* seedlings with (*E*)-2-hexenal induced the transcription of several genes involved in the plant’s defence response including LOX and PAL^29^. Furthermore, exposure to *(Z*)-3-hexenol led to a higher production of VOCs in tomato^30^. The same volatile has recently been shown to be taken up by tomato plants and to be transformed into a glycoside that is toxic to herbivores^31^. In maize, the role of GLVs is more complex. At least three GLVs, (*Z*)-3-hexenal, *(Z*)-3-hexen-1-ol and *(Z*)-3-hexenyl acetate, have been identified to prime inducible production of sesquiterpenes and JA^7^. Also, (*Z*)-3-hexenol has been reported to induce HIPV emission, an effect which was enhanced by simultaneous ethylene exposure^32^. However, in another study, exposure to (*Z*)-3-hexenol led to an increased production of (*Z*)-3-hexenyl acetate and methyl salicylate, but not sesquiterpenes^33^. One possible reason why the role of various HIPVs as volatile priming signals has remained unclear is that in most studies, healthy plants were supplemented with synthetic volatiles, a procedure that does not adequately mimic the precise timing and concentrations of HIPV emissions in nature. The use of ‘deaf’ and ‘mute’ plants has therefore been advocated as a complementary approach to study volatile plant–plant communication^6^. Using this method, it was found that neither GLVs nor terpenoids prime the expression of defence genes in *Nicotiana attenuata*^34^. As altering the capacity of plants to produce HIPVs may lead to unwanted pleiotropic effects, we propose a combination of plant manipulation and synthetic HIPV supplementation as a way forward to compensate for some of the major limitations of each individual approach.

With the exception of salicylates, aromatic HIPVs have received little attention as potential airborne priming signals. Indole in particular is a promising candidate in this context, as it is produced by a wide variety of plants^1^,^35^,^36^,^37^,^38^,^39^ and specifically released in response to herbivore-elicitors^39^,^40^. Furthermore, indole emission in maize peaks about 2 h before to emission of sesquiterpenes^41^, which could enable it to act as a fast and reliable synergistic factor in within-plant induced defence signalling. In maize, indole is produced from indole-3-glycerol phosphate and is channelled into different pathways. First, indole can be formed by the tryptophan synthase-α subunit, which channels it directly to the tryptophan synthase-β subunit for further conversion into the essential amino acid tryptophan^40^,^42^. Second, it can be produced by the BX1 enzyme as an intermediate in the production of benzoxazinoids, a class of non-volatile defensive secondary metabolites of the grasses^43^. Finally, indole can be formed by the indole-3-glycerol phosphate lyase (IGL), which subsequently releases it as a volatile^44^. The *Igl* gene is known to be induced by herbivory, the insect-derived elicitor volicitin and methyl jasmonic acid treatment^40^,^44^. Recently, we isolated an *igl*-mutant in a *bx1* mutant background^45^. This double mutant does no-longer release indole upon herbivore induction. Here, we use these genetic resources to test whether indole is involved in HIPV priming. By exposing maize plants to herbivore-induced volatiles of *igl* mutants or WT plants and to synthetic indole released from dispensers at physiologically relevant concentrations, we show that volatile indole serves as an essential within-plant and plant–plant priming signal in maize.

## Results

### Induced indole emission precedes the release of other HIPVs

**Figure 1:**
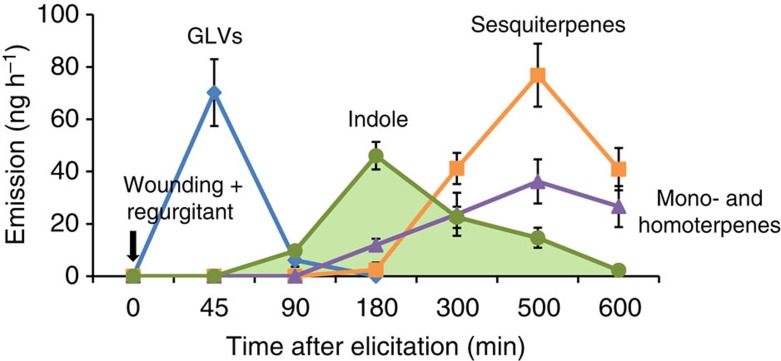
Herbivory-induced indole is released before terpenoids in maize. Two-week-old maize plants (var. Delprim) were induced by scratching the leaf surface and applying 10 μl of *Spodotera littoralis* regurgitant. Five major families of VOCs were induced: green leaf volatiles (GLVs; (*Z*)-3-hexenal, (*Z*)-3-hexen-1-ol, (*Z*)-3-hexenyl acetate)); monoterpenes (Linalool), homoterpenes (3*E*)-4,8-dimethyl-1,3,7-nonatriene (DMNT), (3*E*,7*E*)-4,8,12-trimethyl-1,3,7,11-tridecatetraene (TMTT)); sesquiterpenes ((*E*)-β-caryophyllene, (*E*)-α-bergamotene, (*E*)-β-farnesene) and aromatic compounds (Indole; *n*=6). Error bars correspond to standard errors (±s.e.).

In order to be effective, a within-plant priming signal should be emitted rapidly and specifically upon herbivore attack. To identify airborne priming candidates from herbivore-attacked maize, we artificially damaged three leaves of 10-day-old maize seedlings (hybrid ‘Delprim’) and applied *Spodoptera littoralis* (Lepidoptera: Noctuidae) regurgitant on the scratched leaves. We then collected the HIPVs emitted over a period of 10 h. We found that GLVs are emitted within minutes after herbivore damage. Indole emission started 45 min after elicitation and reached a peak at 180 min. Terpenoid emission started 180 min after elicitation (Fig. 1). These results confirm that indole emission in maize precedes the release of other HIPVs by more than 2 h (ref. 41). Together with the fact that indole is induced in a highly herbivore-specific manner^39^,^40^, this result led to the hypothesis that indole may be involved in airborne priming of terpenoids.

### Synthetic indole primes plants for HIPV release

**Figure 2:**
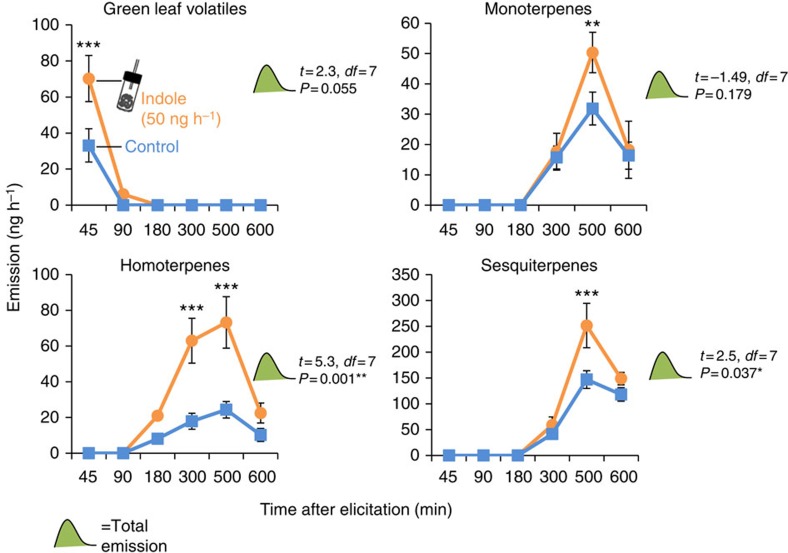
Exposure to volatile indole induces HIPV priming. Hybrid maize seedlings (var. Delprim) were exposed to control- or indole-releasing dispensers for 12 h. They were then elicited by wounding and application of *Spodotera littoralis* regurgitant and placed into clean odour vessels. HIPVs were collected for 600 min. The graphs show the total emissions of four major families of HIPVs for control- and indole-exposed plants at different times after elicitation: green leaf volatiles, monoterpenes, homoterpenes and sesquiterpenes. Asterisks indicate statistical differences between control- and indole-exposed plants (Holm-Sidak *post hoc* tests, *P<0.05, **P<0.01, ***P<0.001, *n*=4–5). *T*-values (*t*), *P*-values (*P*) and residual degrees of freedom (*df*) are shown for *t*-tests comparing total emissions between treatments. Error bars correspond to standard errors (±s.e.). For individual volatiles, see Supplementary Fig. 2.

As a first test of the above hypothesis, we exposed seedlings of the maize hybrid Delprim to control or indole dispensers releasing indole at a physiological dose of 50 ng h^−1^ (Supplementary Fig. 1) and then subjected them to an elicitation treatment as described above. HIPV emissions were measured at different intervals over a period of 10 h after elicitation. Indole exposure itself did not induce the release of volatiles. However, upon elicitation treatment, the release of GLVs, including (*Z*)-3-hexenal, (*Z*)-3-hexen-1-ol and (*Z*)-3-hexenyl acetate from fresh wounds was significantly enhanced in indole-exposed plants (Fig. 2 and Supplementary Fig. 2, Holm-Sidak *post hoc* test: *P*<0.05). Five hours after elicitation, indole-exposed plants also started to emit higher amounts of mono-, homo- and sesquiterpenes (Fig. 2), including linalool, DMNT, TMTT, *(E*)-β-caryophyllene, (*E*)-α-bergamotene and (*E*)-β-farnesene (Supplementary Fig. 2). The total emission of homo- and sesquiterpenes over the whole sampling period was significantly enhanced through indole exposure (Student’s *t*-test: *P*<0.05).

### Indole biosynthesis is required for within-plant priming

**Figure 3:**
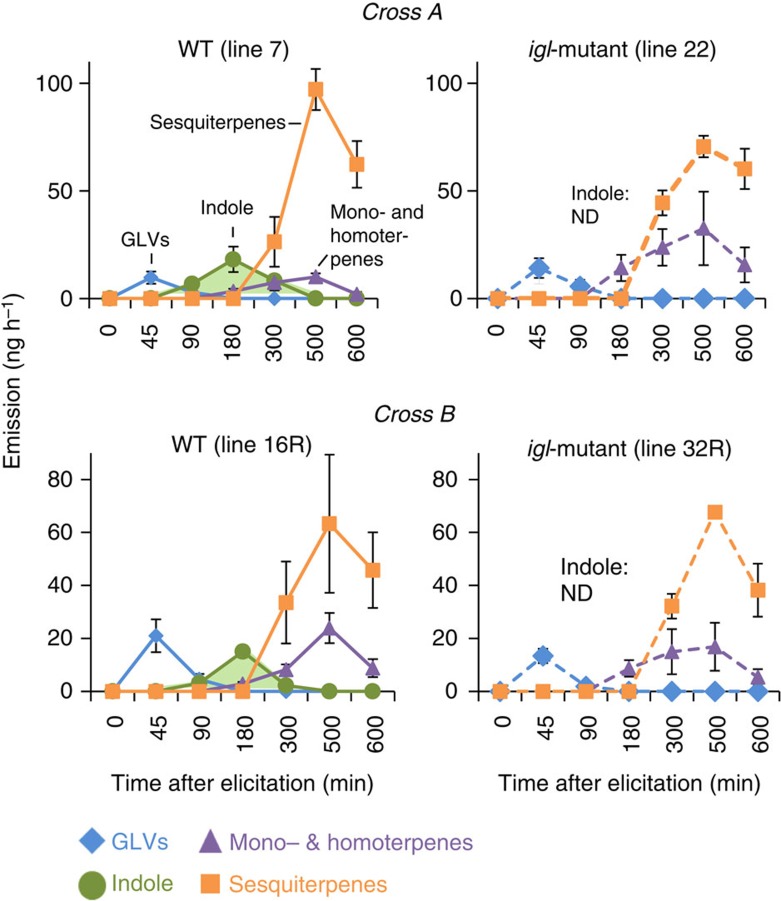
*Igl* mutants are defective in herbivory-induced indole emission. Elicitation treatment was performed by scratching three leaves and applying 10 μl of *Spodotera littoralis* larvae regurgitant to the scratched leaf areas. The graphs show the total emissions of five major families of HIPVs for *Igl*-wild-type and *igl*-mutant plants from cross A and B at different times after elicitation: green leaf volatiles, monoterpenes and homoterpenes, aromatic compounds (indole) and sesquiterpenes. Data correspond to controls of Fig. 4. *Igl* mutants did not emit any indole (green; ND, not detected), but released the other volatile classes in comparable amounts to WT plants (*n*=4). Error bars correspond to standard errors (±s.e.). For individual compounds, see Supplementary Figs 3 and 4.

**Figure 4:**
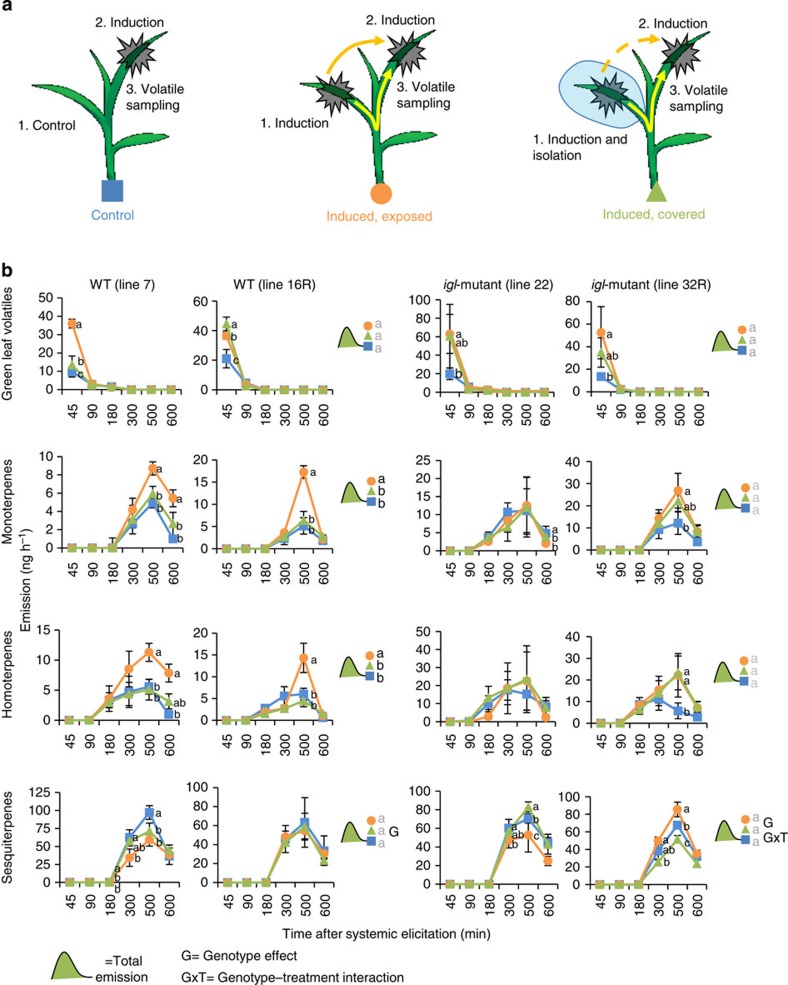
Indole is required for within-plant priming. (**a**) Setup used for experiment. Before the elicitation treatment, the first leaf of each plant was either left intact, induced by scratching and applying *Spodoptera littoralis* regurgitant, or similarly induced and placed in a Teflon bag for 12 h. The second leaf of all plants was then wounded 12h later and volatiles were collected for 600 min. (**b**) Sums of volatiles of the four major families of HIPVs released from WT and *igl* mutant plants are shown: green leaf volatiles, monoterpenes, homoterpenes and sesquiterpenes. Different letters indicate significant differences between treatments (Holm-Sidak *post hoc* tests, *P*<0.05; *n*=4). Error bars correspond to standard errors (±s.e.). For individual compounds, see Supplementary Figs 5 and 6.

To further investigate the role of indole in plant priming, we used *igl* mutant plants in a *bx1* mutant background^46^. The double mutant plants are impaired in the emission of indole, whereas the *bx1* single mutant plants release indole at wild-type levels (Fig. 3). The activity of the *Bx1* gene varies considerably across maize lines^47^, and includes naturally inactive alleles^42^. Consequently, the use of a *bx1* mutant background enabled us to assess the role of IGL-produced indole without potential interference from other sources of free indole. First, we confirmed that *igl* mutants still release all other classes of volatiles in comparable amounts as plants carrying a wild-type *Igl* allele (WT). No systematic quantitative and qualitative differences were found between herbivore-induced volatile blends of WT and *igl* mutants (Fig. 3 and Supplementary Figs 3 and 4). To investigate whether indole is required for systemic priming in unharmed tissues of attacked plants, the first true leaf of *igl* mutant and WT seedlings was either left intact or subjected to elicitation treatment by mechanical wounding and application of oral caterpillar secretions. A subset of the elicited emitter leaves was then wrapped in a small Teflon bag that was sealed around the base of the leaf to minimize HIPV contact of undamaged systemic tissues. All plants were then placed in glass bottles and exposed to a continuous clean airflow of 0.3 l min^−1^ to prevent non-physiological build-up of HIPVs and to isolate the headspace of the different plants. Twelve hours later, all plants were challenged with a second elicitation treatment of leaf 2, after which HIPV emissions were measured at different time points (Fig. 4). During these volatile collections, all the first true leaves were enclosed in a clean Teflon bag to ensure that only volatiles from the second elicitation treatment were captured. No HIPVs except GLVs were detected at the beginning of the second elicitation treatment, indicating that 12 h after elicitation, there was no systemic release of HIPVs induced by the first elicitation treatment anymore^48^. Throughout the sampling period, indole-competent plants that were previously exposed to their own induced headspace released significantly higher total amounts of mono- and homoterpenes than genetically similar plants that were exposed to constitutive volatiles (Fig. 4, Holm-Sidak *post hoc* tests: *P*<0.05). These differences were mainly driven by linalool and DMNT (Supplementary Figs 5 and 6). When volatile exposure was interrupted with a Teflon bag, the systemic priming effect of mono- and homoterpenes disappeared, indicating that within-plant systemic priming of HIPVs in maize partially depends on previous HIPVs exposure. Moreover, indole-deficient mutant plants released the same total amounts of mono- and homoterpenes, irrespective of previous exposure to their own (indole-free) HIPV blend (Fig. 4), showing that indole is required for within-plant priming of the above volatiles.

The within-plant priming response of GLVs followed a different pattern than mono- and homoterpenes. Although the total amount of released GLVs was not influenced by any of the priming treatments (two-way analysis of variance (ANOVA), *P*>0.05), GLV release 45 min after elicitation was consistently enhanced by previous defence elicitation (Fig. 4b). Additional self-HIPV exposure in indole-producing WT line 7 resulted in augmented GLV emission from leaf 2 compared with un-elicited control treatment and elicitation of a covered first leaf. However, this airborne priming by GLV was not apparent in line 16R, where self-HIPV exposure resulted in a similar augmentation of GLV emission in leaf 2 as was found after covered elicitation treatment of leaf 1 without HIPV exposure. Previous elicitation primed GLV emissions in the igl mutants independently of HIPV exposure, suggesting that vascular signals are involved in their systemic priming. However, the HIPV-specific modulations were absent, indicating that indole modulates GLV-priming in a genotype-specific manner. The total amounts of sesquiterpenes did not differ between treatments and genotypes (two-way ANOVA, *P*>0.05). In WT line 7, self-HIPV exposure supressed sesquiterpene emissions at 300 min post elicitation of leaf 2, whereas in WT line 16R did not display differences in induced sesquiterpene emissions of leaf 2 between pre-treatments. The indole mutant line 22 responded similar to its WT counterpart. Conversely, mutant line 32R showed suppressed sesquiterpene emission from leaf 2 after pre-elicitation of leaf 1 without HIPV exposure, but it showed augmented sesquiterpene emission from leaf 2 upon self-HIPV exposure from leaf 1 (Fig. 4b).

**Figure 5:**
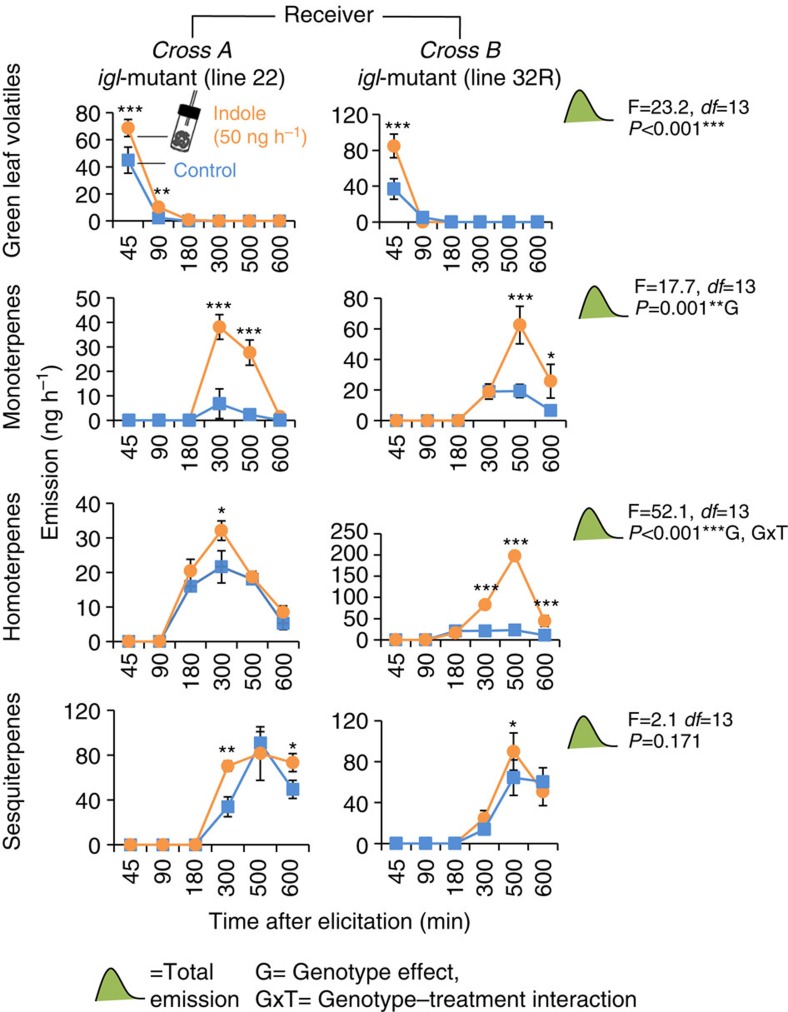
Indole exposure restores within-plant priming in *igl*-mutants. Maize seedlings were exposed to control or volatile indole dispensers for 12 h. They were then elicited by wounding and application of *S. littoralis* regurgitant and placed into clean vessels. HIPVs were collected for 600 min. The graphs show the total emissions of four major families of HIPVs for control- and indole-exposed plants at different times after elicitation: green leaf volatiles, monoterpenes, homoterpenes and sesquiterpenes. Asterisks indicate statistical differences between control- and indole-exposed plants (Holm-Sidak *post hoc* tests, *P<0.05, **P<0.01, ***P<0.001, *n*=4–5). F-values (F), *P*-values (*P*) and residual degrees of freedom (*df*) are shown for ANOVAs comparing total emissions between treatments and genotypes. Error bars correspond to standard errors (±s.e.). For individual compounds, see Supplementary Figs 7 and 8.

Exposure of *igl* mutants to synthetic indole restored augmented emission of mono- and homoterpenes and primed GLV emission (Fig. 5, supplementary Figs 7 and 8). Sesquiterpene emissions were also slightly enhanced. Taken together, these results demonstrate that indole is required for HIPV-induced priming of herbivore-elicited emissions of mono- and homoterpenes by HIPVs. The strength and direction of indole-dependent priming of GLVs and sesquiterpenes, on the other hand, vary substantially between genotypes.

To assess whether previous isolation of leaf 1 with a Teflon bag alters the subsequent inducibility of volatile emissions, we performed an additional control experiment with the hybrid Delprim. We detected no significant effects of bagging on monoterpenes and sesquiterpenes. However, induced (*Z*)-3-hexenyl acetate emission was slightly enhanced, and DMNT emission was suppressed (Supplementary Fig. 9), indicating that the differences in GLV and monoterpene emissions between bagged and non-bagged plants within genotypes in Fig. 4 should be interpreted with care.

### Indole primes neighbouring plants

**Figure 6:**
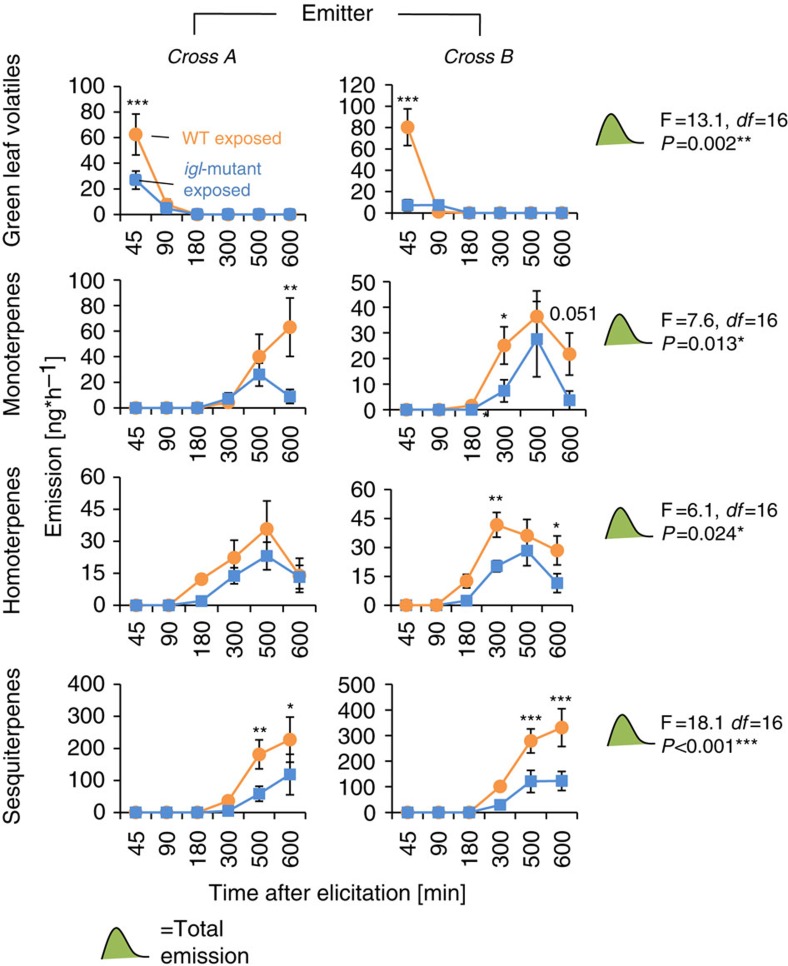
Indole-containing volatile blends enhance priming of neighbouring plants. Hybrid maize seedlings (*var.* Delprim) were exposed to indole-deficient *igl*-mutant or *Igl*-wild-type infested plants for 12 h. They were then elicited by wounding and application of *Spodoptera littoralis* regurgitant and put in clean vessels. VOCs were collected for 600 min. HIPV emission of hybrid maize plants exposed to induced volatiles from *igl*-mutant (Cross A: line 22; Cross B: line 32R) or *Igl*-wild-type plants (Cross A: line 7; Cross B: line 16R) are shown. Sums of volatiles of the four major families of HIPVs are presented: green leaf volatiles, monoterpenes, homoterpenes and sesquiterpenes. For individual compounds, see Supplementary Figs 12 and 13. Asterisks indicate statistical differences between release rates from control- and indole-exposed plants (Holm-Sidak *post hoc* tests, *P<0.05, **P<0.01, ***P<0.001, *n*=5). F-values (F), *P*-values (*P*) and residual degrees of freedom (*df*) are shown for ANOVAs comparing total emissions between treatments and genotypes. Error bars correspond to standard errors (±s.e.). For individual compounds, see Supplementary Figs 10 and 11.

To test whether volatile indole also acts as a priming agent between plants, we exposed healthy Delprim plants to HIPVs from *igl*-mutant and WT lines. HIPV production was on average two to three times higher in WT exposed than in *igl*-mutant exposed plants: At different time points after elicitation, the emission of GLVs, mono-, homo- and sesquiterpenes were significantly enhanced in WT exposed seedlings (Fig. 6, Supplementary Figs 10 and 11), demonstrating that maize plants increase their defensive responsiveness upon perception of herbivore-induced indole from neighbouring plants. The total amounts of all four HIPV classes were also significantly enhanced. Both genetic backgrounds primed the hybrid Delprim to a similar extent (two-way ANOVAs, genotype effect: *P*>0.05), apart from homoterpenes, for which the difference between priming from the WT and mutant lines was more pronounced for cross B (two-way ANOVA, *P*<0.05). On an individual basis, the strongest and most consistent priming effects were recorded for (*Z*)-3-hexenal, (*Z*)-3-hexen-1-ol, linalool, (*E*)-α-bergamotene and (*E*)-β-farnesene (Supplementary Figs 10 and 11).

### Indole exposure increases induced phytohormone levels

**Figure 7:**
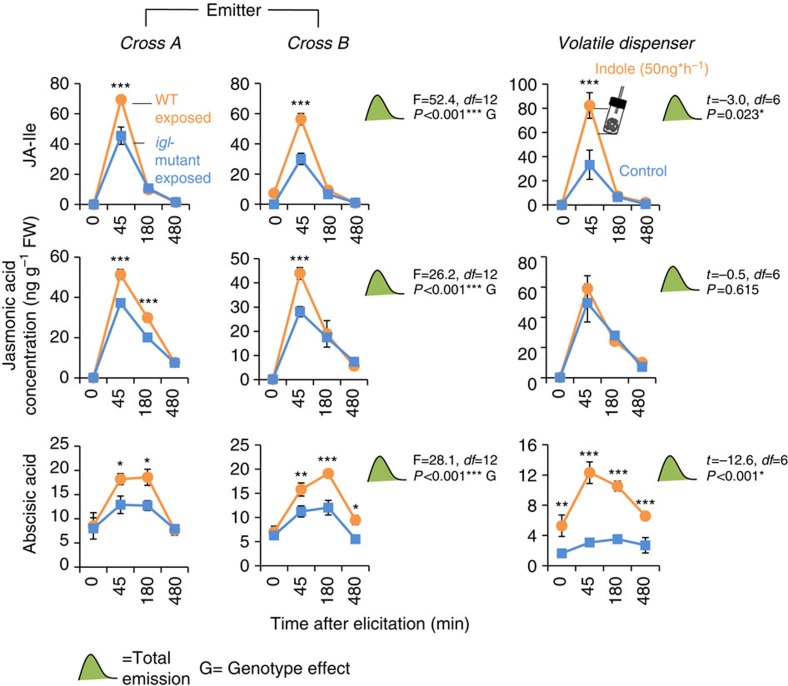
Indole primes jasmonate (JA) and abscisic acid (ABA) production. Hybrid maize seedlings were exposed to dispensers or wild-type or indole mutant plants for 12 h. Leaf material was harvested at 0, 45, 180 and 480 min after elicitation treatment. Asterisks indicate statistical differences in the levels of ABA, JA and jasmonic acid isoleucine (JA-Ile) between control- and indole-exposed plants. (Holm-Sidak *post hoc* tests, *P<0.05, **P<0.01, ***P<0.001, *n*=3–4.) F-values (F), *t*-values (*t*), *P*-values (*P*) and residual degrees of freedom (*df*) are shown for ANOVAs and *t*-tests comparing total production between treatments and genotypes. Error bars correspond to standard errors (±s.e.).

To study the mechanism of the observed indole priming, we quantified induced defensive phytohormones in indole-exposed maize seedlings. Jasmonates in particular are known to regulate HIPV release in maize^49^. Leaves of indole-exposed and control plants were collected at different time points after elicitation treatments in two separate experiments. First, Delprim seedlings were exposed to volatiles from induced *igl*-mutant and WT plants. Second, Delprim plants were exposed to control or indole-releasing dispensers. Delprim seedlings exposed to indole-producing or indole-deficient plants had the same constitutive levels of abscisic acid (ABA), JA and JA conjugated with isoleucine (JA-Ile). However, upon elicitation, the levels of all three hormones increased more strongly in indole-exposed seedlings (Fig. 7). Forty-five minutes after elicitation, JA and JA-Ile levels were 50% higher in indole-exposed plants. Calculated total amounts of hormones across the sampling period were also significantly higher in WT exposed plants. WT and mutants of both crosses elicited similar responses, although total hormone concentrations in Delprim plants that had been exposed to plants from cross A were slightly higher than Delprim plants that had been exposed to plants from cross B. Similar priming effects were obtained by exposing seedlings to realistic concentrations of synthetic indole (Fig. 7), with the exception of JA, which did not respond to indole alone.

### Specificity of volatile priming by indole

To assess whether indole can prime other plant species apart from maize, we performed an experiment in which we exposed cotton (*Gossypium hirsutum*) and cowpea (*Vigna unguiculata*) to indole dispensers (50 ng h^−1^) for 12 h, followed by an induction treatment (wounding and *S. littoralis* regurgitant) and volatile sampling between 180 and 630 min post induction. Maize seedlings were included as a positive control. To standardize data analysis, volatile profiles of the different species were normalized with XCMS software, after which the identified compounds were analysed by principal component analysis (PCA) and ANOVA. For all three species, we detected typical HIPV features, including DMNT in cowpea and α-pinene and (*E*)-β-caryophyllene in cotton. A clear indole priming effect was observed for maize: The unsupervised data mining approach confirmed that indole exposure enhances the induced emission of mono-, homo- and sesquiterpenes (Table 1), and the two different treatments were separated along the first axis of the PCA (Supplementary Fig. 12). By contrast, no differentially regulated features were detected in cowpea, for which the PCA did not separate between both treatments (Supplementary Fig. 13). In cotton, we identified two features which were significantly induced through indole exposure. No clear database matches for the two peaks were found. Furthermore, PCA failed to clearly separate indole exposure and control treatments (Supplementary Fig. 14). These results indicate that indole may boost the production of selected volatiles in other plants, but the coordinated priming response of a metabolically broad range of terpenoids by indole seems to be specific for maize.

## Discussion

Several studies have shown that HIPVs prime for direct and indirect plant defences^7^,^8^,^16^,^28^,^50^. Yet, the identity of the volatile messengers has remained elusive in most cases. The current study reveals an important role of indole in HIPV-induced priming via several lines of evidence: (i) Synthetic indole primes volatile release upon simulated herbivory. (ii) Systemic within-plant priming is absent in indole-deficient *igl*-mutant plants and can be restored by applying synthetic indole. (iii) Exposure to synthetic indole or indole-containing HIPVs primes volatile release in neighbouring plants. (iv) Exposure to indole or indole-containing volatile blends primes plants for the production of defensive phytohormones.

Earlier studies have proposed other HIPVs as priming signals. Among them, GLVs have been documented repeatedly to possess signalling capacity^7^,^27^,^28^,^33^,^51^. However, the relative contribution of GLVs compared with other potential HIPV signals remains unclear. Kost and Heil^28^ showed that lima bean plants increase production of extra floral nectar after exposure to naturally emitted GLVs and (*Z*)-3-hexenyl acetate. However, the weaker effect of the synthetic VOCs blend compared with plant-derived HIPVs indicated that other constituents of the HIPV blend could also have contributed to the priming. Because GLVs are generally emitted after physical leaf damage, they are unreliable signals for an impending herbivore attack. Herbivore-specific volatiles like indole are therefore likely to complement and/or enhance the information value of GLVs. In the case of maize, our experiments consistently indicate that indole is required for systemic priming. However, we cannot exclude that GLVs and other volatiles enhance indole-mediated signalling. We found that volatile indole is perceived not only by the emitting plant itself, but also by neighbouring plants. Target plants previously exposed to infested WT plants produced larger amounts of volatile compounds shortly after an herbivore attack than plants exposed to infested *igl*-mutant plants. Similar results were obtained when we exposed target plants to synthetic indole alone. This shows that volatile indole alone is sufficient to induce priming in receiving plants. Given that other volatiles may also possess priming activity^7^,^27^,^28^,^33^,^51^, the within-plant signalling role of indole that leads to enhanced emission of GLVs and terpenoids may trigger a cascading effect, which would further boost the emitted volatile blend of neighbouring plants that have perceived indole as an early warning.

Previous studies have shown that in the case of limited or absent vascular connections, HIPVs are sufficient to reduce herbivore feeding^13^,^16^. Here we show that the systemic priming effect is absent when volatile exposure is blocked, further confirming that within-plant systemic priming of HIPVs requires previous HIPV exposure. These results are in accordance with a model that combines vascular and volatile signalling as proposed by Heil and Ton^9^. They suggest that priming involves a two steps regulatory system where airborne signals sensitize distal plant parts for a second vascular signal upon herbivore attack. An interesting observation in this context is that GLVs and sesquiterpenes responded in a genotype-specific manner in the within-plant priming experiments. Depending on the genetic background, indole either enhanced or supressed within-plant priming of these two volatile classes, suggesting that yet unknown factors determine the direction of the indole effect for these classes of volatiles. It is tempting to speculate that potential vascular signals alter the distal response of a receiving leaf in a genotype-specific manner, which could add another layer of regulation to within-plant priming that enables plants to distinguish between self and non-self HIPVs.

We also noted differences in priming patterns for individual volatile compounds between experiments. Overall, all major maize volatiles showed positive responses in more than one experiment. Furthermore, we did not find any individual volatile that was consistently and significantly suppressed by indole. The most consistently primed volatiles were the monoterpene linalool, which was significantly primed in all eight experiments, followed by the homoterpene DMNT and the GLV (*Z*)-3-hexenyl acetate, which were significantly primed in 80% of all trials. (*Z*)-3-Hexenal, (*Z*)-3-hexen-1-ol, (*E*)-α-bergamotene and (*E*)-β-farnesene were primed in more than 50% of all experiments, whereas TMTT and (*E*)-β-caryophyllene were only primed sporadically. We propose two mutually non-exclusive hypotheses that could account for the variability at the individual volatile level. First, genetic variation in the emission of HIPVs other than indole and in the capacity to perceive indole may have contributed to this variation. This hypothesis is supported by the fact that the hybrid Delprim responded more consistently to indole exposure than the different mutant crosses. Second, environmental variation may have altered the integration of the indole signal into the regulatory network that governs HIPV production and release. Further research will be necessary to evaluate the relative importance of plant genetics and the environment on indole-dependent priming.

The elucidation of the elements of defensive signalling that are enhanced through priming is an important next step to understand the mechanism behind HIPV-mediated priming. The role of JA in HIPVs emission in maize has been well documented^49^,^52^,^53^, and previous studies showed the importance of the octadecanoid pathway in the GLV-mediated priming^7^,^27^,^33^. Here, we found that exposure of plants to indole or indole-containing HIPVs enhances the herbivore-induced production of the phytohormones ABA, JA and JA-Ile. We therefore propose that indole acts upstream of defence hormonal signalling to increase the production of herbivore-induced HIPVs. The fact that indole-containing HIPVs, but not indole alone, led to priming of the pro-hormone JA suggests that full jasmonate priming depends on the interplay between indole and indole-enhanced HIPVs. As indole alone was sufficient to prime the actual hormone JA-Ile, the significance of the JA phenotype remains to be determined. Another intriguing questions in this context is whether and how plants perceive indole. In the bacterium *Stigmatella aurantiaca*, a pyruvate kinase was identified as a putative indole receptor^54^, and it is possible that plants employ similar proteins to detect indole in their environment. By using volatile priming as a rapid and simple readout, forward and reverse genetic approaches could be employed to identify potential indole receptors in maize. Another mechanism by which indole could act as a priming agent is through its uptake and metabolization to other bioactive products. IGL-derived indole may be incorporated into the biosynthesis of non-volatile benzoxazinoids^55^, which by themselves can act as defensive inducers *in planta*^46^,^56^. It is therefore possible that indole-derived metabolites trigger the actual priming response in maize.

Many plant species release indole upon herbivore damage^57^, prompting the question whether the volatile may be a general defence priming signal in nature. Our experiments suggest, however, that other plants are much less responsive to indole than maize, as no significant priming was observed in cowpea, and only weak responses were detected in cotton. The fact that indole priming is restricted to a few plant species would be compatible with the notion that priming signals have originally involved as private messages that allowed plants to warn their own non-attacked tissues^10^ or their close kin^58^ from incoming attack. Induced volatile perception by plants occurs in nature^13^,^15^. As herbivore-infested maize plants release significant amounts of indole in the field^59^, it is plausible that the volatile also primes plant tissues of conspecific neighbouring plants under these conditions. Actual field experiments will be necessary to confirm this hypothesis.

In conclusion, we propose that indole is a reliable and effective signal for priming because its release is greatly enhanced by herbivory as compared with mere mechanical damage^40^ and it is released faster than other inducible volatiles^41^. The presented findings are likely to facilitate the unravelling of the mechanisms of priming and to help testing its ecological relevance.

## Methods

### Plant cultivation

The maize lines 22 (*igl.bx1*), 7 (*Igl.bx1*) (genetic background A) 32R (*igl.bx1*) and 16R (*Igl.bx1*) (genetic background B) were obtained as previously described^46^. Briefly, reciprocal crosses between the reference *bx1* mutant^60^ and the homozygous Mu-insertion mutant of *Igl* were performed. Two pairs of individual F1 plants of both crosses were intermated to generate independently homozygous single and double mutants (genetic background A and B). As loci unlinked to *Igl* and *Bx1* will segregate in the mutant progeny, analysing pairs of plants from two pedigrees allows to recognize background effects on volatile emission. Seeds of the hybrid Delprim, which are homozygous for both *Bx1* and *Igl* alleles, were obtained from Delley Semences et Plantes SA., Delley DSP, Switzerland. All maize lines were grown individually in plastic pots (10 cm high, 4 cm diameter) with commercial potting soil (Aussaaterde, Ricoter, Aarberg, Switzerland) and placed in a climate chamber (23 °C±2 °C, 60% relative humidity, 16:8 h light/dark, 50,000 lm m^−2^). Maize plants used for the experiments were 10- to 12-day old and had three fully developed leaves. The evening before the experiments, plants were transferred and kept under laboratory conditions (25±2 °C, 40±10% relative humidity, 16 h light/8 h dark, and 8,000 lm m^−2^).

### Within-plant priming with supplementation of synthetic indole

To test whether volatile indole is a key compound in within-plant priming in maize, we exposed different maize lines to synthetic indole for 12 h. Delprim plants and *igl*-mutant plants (lines 22 and 32R) were then subjected to an elicitation treatment and put into clean odour vessels in the presence of a control or indole dispenser. Plants were connected to a multiple air-delivery system via Teflon tubing. This system consisted of a central wooden tray with 6 or 12 glass odour source vessels^8^, a metal frame with eight neon tubes (four Osram 18W/21-810 alternated with four Sylvania Gro-Lux F18W/GRO-T8), and one or two manifolds with six flow metres (Aalborg Instruments & Controls), each followed by charcoal filters and water bubblers filled with MilliQ-water (Model VCS-HADS-6AF6C6B; ARS Analytical Research System). The elicitation treatment was performed by scratching two leaves over an area of approximately 1 cm^2^ on both sides of the central vein with anatomical forceps (stainless steel, 14.5 cm; *n*=4). Then 10 μl of *Spodoptera littoralis* regurgitant were applied over the scratched leaf areas. Regurgitant had been previously collected from fourth instar *S. littoralis* that had been feeding on maize leaves for 24 h and was then stored at −80 °C until use. Dispensers consisted of 2 ml amber glass vials (11.6 × 32 mm^2^; Sigma-Aldrich) containing 20 mg of synthetic indole (>98%, GC, Sigma-Aldrich). The vials were sealed with a PTFE/rubber septum pierced by a Drummond 1 μl micro-pipette (Drummond, Millan SA) in black polypropylene cap. This device allowed the constant release of volatile indole. The length of the pipette was calibrated to release 50 ng h^−1^ of indole, which corresponds to the amount emitted by WT plants (*Zea mays* cv. Delprim). Control dispensers consisted of empty glass vials. Vials were prepared the day of the experiment. VOCs were collected for 600 min following elicitation.

### Within-plant priming in *igl*-mutant and WT plants

To confirm the specific role of indole as a within-plant priming agent, we performed an experiment using *igl*-mutant and WT plants of both genetic backgrounds. Plants used for this experiment consisted of *igl*-mutant plants (lines 22 and 32R) and WT plants (line 7 and 16R). Plants were submitted to three different treatments (*n*=4). Two groups were subjected to an elicitation treatment as previously described. For one of these groups, a Teflon bag (8 × 3 cm^2^) was placed around the wounded leaf in order to prevent VOCs to act as a volatile priming signal. The second group was wounded and left without Teflon bag. The last group was left undamaged. All plants were put into clean odour vessels. Twelve hours later (6 h light/6 h dark), the second leaf of all groups was subjected to an elicitation treatment as previously described. A new Teflon bag was put on the first leaf of each plant in order to prevent the collection of volatiles from the first leaf. All plants were put in clean odour vessels and connected to the multiple air-delivery system. VOCs were collected for 600 min at intervals as described below. In an additional control experiment, we bagged the second leaf of Delprim plants for 12 h, removed the bag and induced previously bagged or non-bagged plants on leaf 3 as described, followed by volatile collections in 2 h intervals for a total of 8 h (*n*=3).

### Plant–plant communication between *igl*-mutants and WT plants

To test whether volatile indole could also prime neighbouring plants, we exposed healthy Delprim plants to VOCs from infested *igl*-mutant plants or WT plants of both genetic backgrounds (*n*=4). Source and target plants were individually introduced into glass vessels. Source plants consisted of *igl*-mutant plants (lines 22 and 32R) or WT plants (line 7 and 16R). Target plants were either Delprim plants or WT plants (line 7 and 16R). Source plants were infested with 20 first-instar *S. littoralis* larvae that were placed into the whorl of the youngest leaves. The glass vessels with the plants were connected to a multiple air-delivery system via Teflon tubing. Four hours later, target plants in similar vessels were exposed to air from herbivore-infested *igl*-mutant plants; or air from herbivore-infested WT plants at a flow rate of 0.3 l min^−1^. After 12 h of exposure, target plants were subjected to an elicitation treatment as described above and put in clean odour vessels for VOCs collection. VOCs were collected for 600 min.

### Specificity of indole-induced VOC priming

To get first insights into the specificity of indole-dependent priming, we exposed cotton, cowpea and maize seedlings to synthetic indole as described (*n*=3–4). Because of their slower growth, cotton and cowpea seedlings were used for experiments 5 weeks after sowing. All seedlings were then induced by wounding and *S. littoralis* regurgitant as described above. Volatiles were collected for periods of 90 min starting 90 min after elicitation and ending at 630 min. At the end of the experiment, the above ground parts of all plants were harvested, and fresh weight was determined.

### Volatile collection and analysis

VOC collections for all experiments were carried out by using a multiple air-delivery system. Purified air from the system entered the source vessels via Teflon tubing at a rate of 1.1 l min^−1^ and was pulled out through the Super-Q trap at a rate of 0.7 l min^−1^. Super-Q trap consisted of 7 cm glass tubes in which 25 mg of 80–100 mesh Super-Q adsorbent (Altech) kept in place by one fine mesh metal screen on the one side and a little quantity of fibreglass held by a piece of Teflon (2 mm length) on the other side. In all experiments, a filter was attached to the horizontal port at the top of each odour source vessel. The other ports were sealed with a Teflon-coated septum in the screw cap. A 6-mm inner diameter Tygon tube was connected to each collection trap, through which air was pulled out. Before each experiment, the traps were rinsed with 3 ml of methylene chloride. Super-Q traps from vessels containing elicited plants were collected at 45, 90, 180, 300, 500 and 600 min after elicitation treatment unless described otherwise. Immediately after each collection, traps were exchanged and the volatiles collected on the trapping filters were extracted with 150 μl of methylene chloride and two internal standards (*n*-octane and nonyl-acetate, each 200 ng in 10 μl methylene chloride) were added to these samples. For the analysis, an aliquot of 3 μl was injected on-column with the use of an automated injection system onto an apolar HP-1 capillary column, which was preceded by a deactivated retention gap (10 m, 0.25 mm I.D.) and a deactivated precolumn (30 cm, 0.53 mm I.D.). The columns were housed in a Hewlett Packard model HP 6890 gas chromatograph equipped with a flame ionization detector. The oven was held at 50 °C for 3 min and then programmed at 8 °C min^−1^ to 230 °C, where it was maintained for 9.5 min. Helium was used as carrier gas. HP GC Chemstation software was used to quantify all major compounds based on the known quantity of internal standards, unless stated otherwise. Initial identification of most compounds was based on comparisons of retention times with those from previous studies. Identities were confirmed with the mass spectrometry analysis of some samples, using the same column and temperature programme (Agilent 5973, transfer line 230 °C, source 230 °C, quadrupole 150 °C, ionization potential 70 eV, scan range 0–400 a.m.u.). To obtain an estimate of the different classes of VOC, total amounts of the following compounds were summed up: GLVs: (*Z*)-3-hexenal, (*Z*)-3-hexen-1-ol and (*Z*)-3-hexenyl acetate; monoterpenes: linalool; homoterpenes: 4,8-dimethyl-1,3 (*E*), 7-nonatriene and 4,8,12-tri-methyl-1,3(*E*),7(*E*),11-tridecatetraene; sesquiterpenes: (*E*)-β-caryophyllene, (*E*)-α-bergamotene, (*E*)-β-farnesene. For the specificity experiment, volatile data were processed using GC/Single Quad (centWave) parameters in XCMS online 61. All samples of one species were analysed together. The detected features were corrected for the internal standard signal intensity and plant weights, and the corrected intensities of the different features were summed up for statistical analysis.

### Phytohormone quantification

In order to determine the mechanism of the observed indole priming, we quantified phytohormones in two independent experiments. Delprim plants were exposed to volatile indole or infested *igl*-mutant (lines 22 and 32R) and WT (line 7 and 16R) plants for 12 h as previously described. Leaf material was collected before wounding and 45 min, 3 and 8 h after elicitation treatment. Leaf material (1 cm^2^) adjacent to the wound-site was collected, flash frozen in liquid nitrogen and stored at −80 °C. ABA, JA and JA-Ile were quantified using an ultra-high-pressure liquid chromatography-tandem mass spectrometry (UHPLC/MS-MS). Hundred microgram per samples were extracted in 990 μl of EtOAc: formic acid, 99.5:0.5 (v/v) and 10 μl of an internal standard solution containing isotopically labelled JA and ABA (10 ng ml^−1^). The extracts were centrifuged at 14,000 *g* for 3 min. The supernatant was transferred in a 2-ml Eppendorf tube and 500 μl of EtOAc/formic acid, 99.5:0.5 (v/v) was added to the pellet and the same procedure was repeated. The extracts were then evaporated to dryness and re-suspended in 100 μl of aqueous methanol (50:50 (v/v)). After centrifugation, 5 μl were injected into the UHPLC/MS-MS. The hormones were quantified by calculating a calibration equation obtained by linear regression from five calibration points for each compound. Peak areas of the hormones measured in the samples were normalized to the internal standard before applying the calibration equation.

### Statistical analyses

Differences in HIPV emissions between control- and indole-exposed plants and between mutant and WT plants were assessed using ANOVAs. Repeated measures ANOVAs followed by Holm-Sidak *post hoc* tests were employed to compare treatments across time. Furthermore, volatiles were summed up over time, and total emissions were compared using (i) Student’s *t*-tests for single treatment experiments and (ii) two-way ANOVAs for multifactorial experiments involving different treatments and genotypes. All ANOVAs were performed with SigmaPlot 12.5. Test statistics can be found in the supplementary files (Supplementary Data 1 and 2). PCAs were performed using the FactoMineR package in R 3.1.0 (ref. 62).

## Author contributions

M.E., N.V. and T.C.J.T. designed the experiments. N.V., C.A.M.R. and H.X. performed the experiments. M.E., N.V., C.A.M.R., J.T., M.F. and T.C.J.T analysed data. M.E., N.V. and T.C.J.T. wrote the manuscript.

## Additional information

**How to cite this article:** Erb, M. *et al*. Indole is an essential herbivore-induced volatile priming signal in maize. *Nat. Commun.* 6:6273 doi: 10.1038/ncomms7273 (2015).

## Supplementary Material

Supplementary Figures Supplementary Figures 1-14

Supplementary Dataset 1 Statistical tables for main figures.

Supplementary Dataset 2 Statistical tables for supplementary figures.

## Figures and Tables

**Table 1:** Specificity of indole-primed volatile release.

**Maize**	**Detected features** **94**	**Differential features** **5**	
**Ret. time;** ***m*****/*****z***	**ID**	**Fold change**	***P*****-value**
12.9; 92.1	Linalool	2.5	0.031
13.4; 79.1	DMNT	1.4	0.011
21.2; 77.1	(*E*)-α-Bergamotene	1.3	0.039
21.6; 40.2	(*E*)-β-Farnesene	1.5	0.046
21.6; 105.1	(*E*)-β-Farnesene	1.4	0.024
**Cotton**	**Detected features** **102**	**Differential features** **2**	
			
**Ret. time;** ***m*****/*****z***	**ID**	**Fold change**	***P*****-value**
7.1; 207.1	Unidentified	1.5	0.021
14.9; 73.1	Unidentified	1.9	0.028
**Cowpea**	**Detected features**	**Differential features**	
	**64**	**0**	

DMNT, (3E)-4,8-di-methyl-1,3,7-nonatriene; Ret. time, retention time.

Maize, cotton and cowpea plants were exposed to synthetic indole for 12 h and then wounded and treated with *S. littoralis* regurgitant. Volatiles were collected every 90 min between 180 and 630 min following induction. After automated alignment and feature detection, internal standard signal intensity and plant weight correction, the intensities of the different features were summed up for statistical analysis. Differentially regulated features are shown (*n*=3–4, Student’s *t*-test, *P*<0.05).
