# Supplementary material for: Indole is an essential herbivore-induced volatile priming signal in maize
Source: Nat Commun. 2015 Feb 16;6:6273. doi: 10.1038/ncomms7273 (PMC4339915; doi:10.1038/ncomms7273)
Supplement: Supplementary Figures — 1-14 [file ncomms7273-s1.pdf]

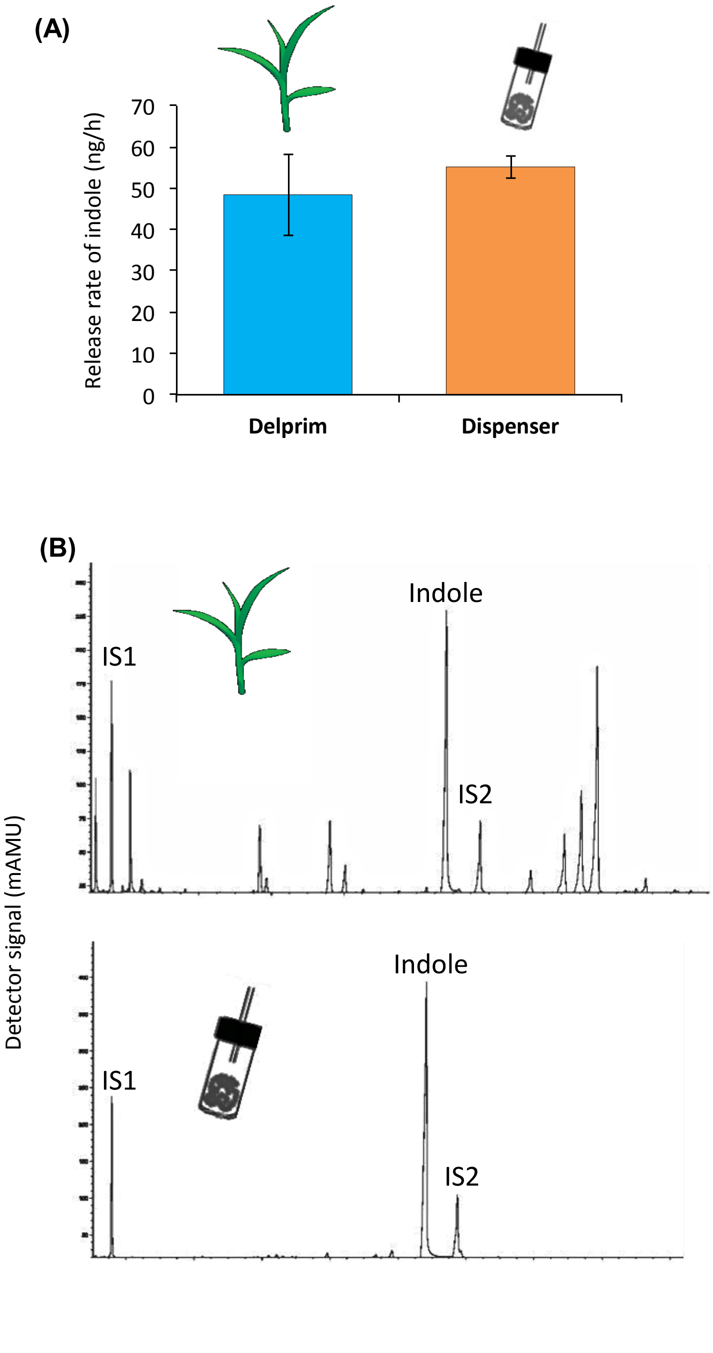

Supplementary Fig. 1: Releasers emit physiologically relevant levels of indole. (A) Release rate of indole from infested Delprim plants and capillary dispensers (n=8). **(B)** GC/FID chromatograms showing HIPVs emitted by Delprim after one day of *S. littoralis* infestation (top) and an indole capillary dispenser (bottom). IS= Internal standard.

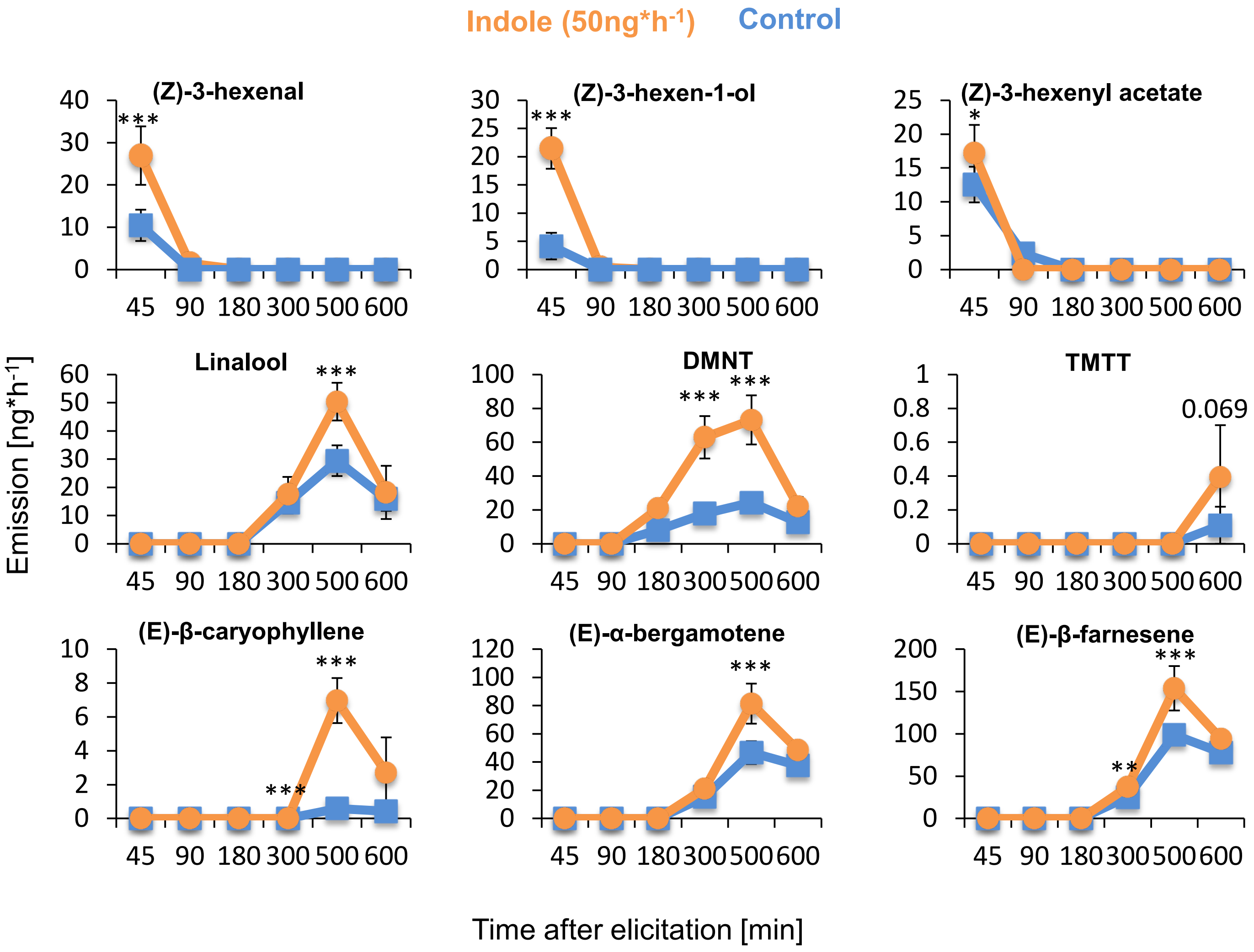

Supplementary Fig. 2: Individual HIPVs of experiment shown in figure 2. For experimental details, see figure 2

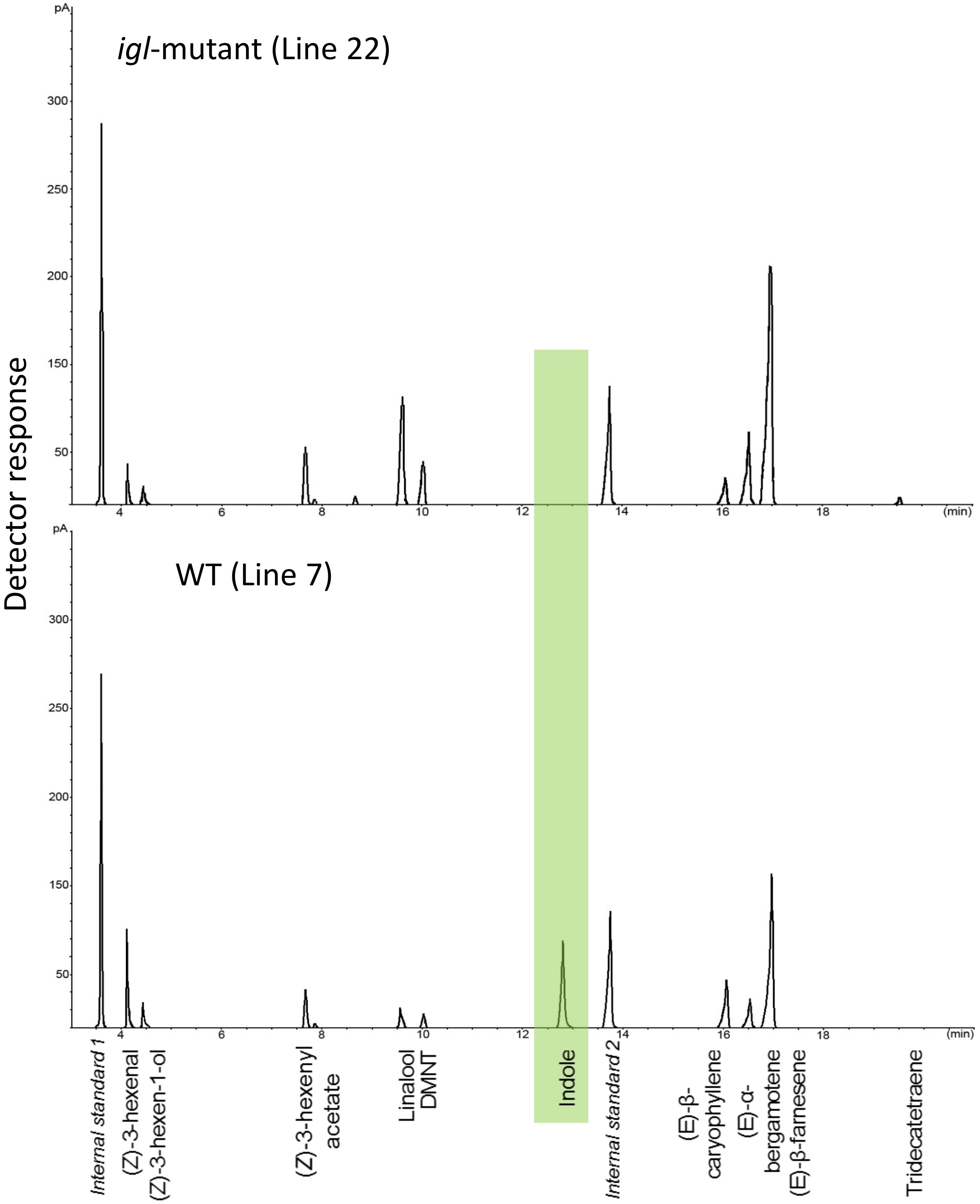

Supplementary Fig. 3: GC/FID Chromatograms of volatile release of indole-deficient mutants (line 22) and wild type (line 7) plants from cross A 8 h after elicitation.

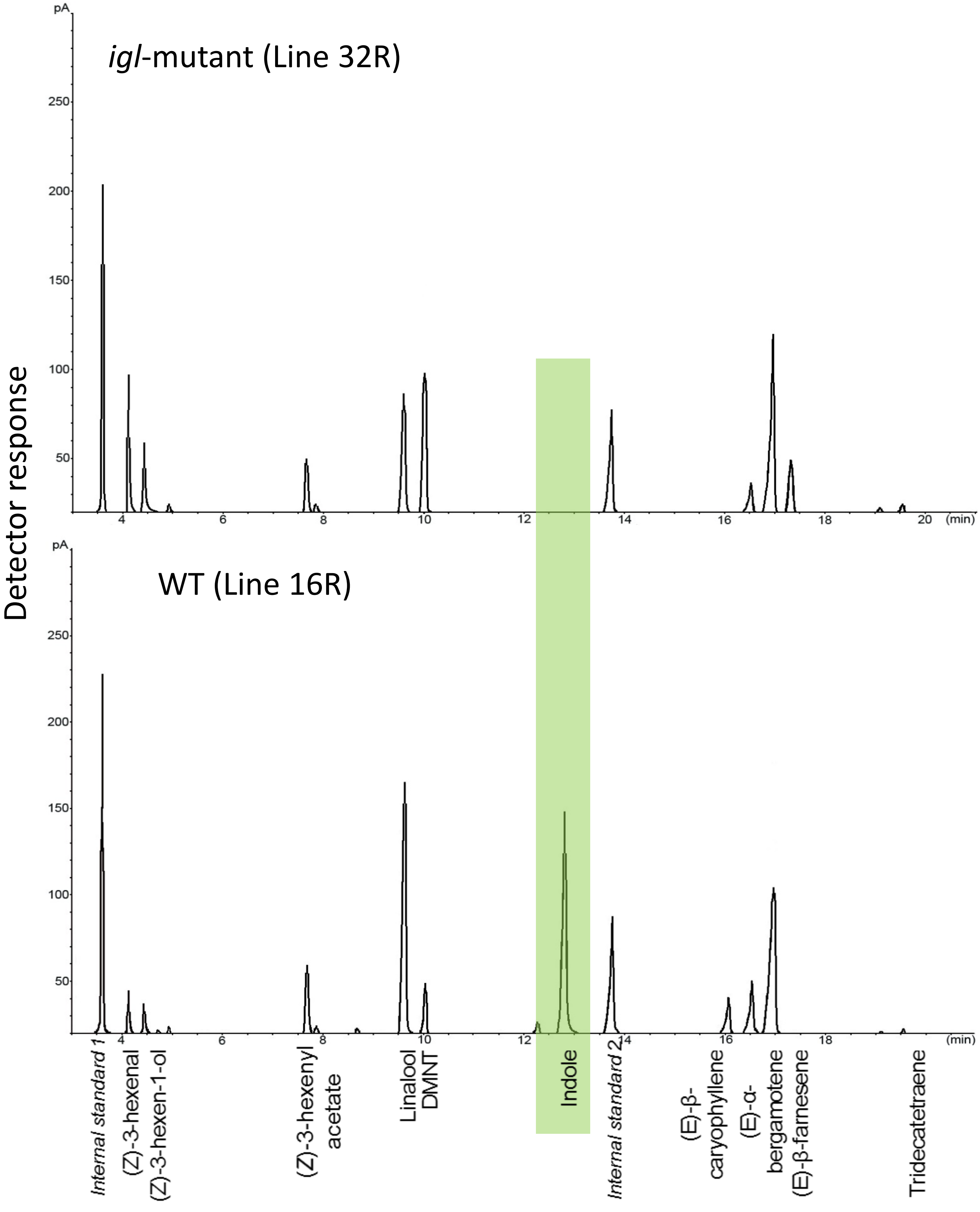

Supplementary Fig. 4: GC/FID Chromatograms of volatile release of indole-deficient mutants (line 32R) and wild type (line 16R) plants from cross A 8 h after elicitation.

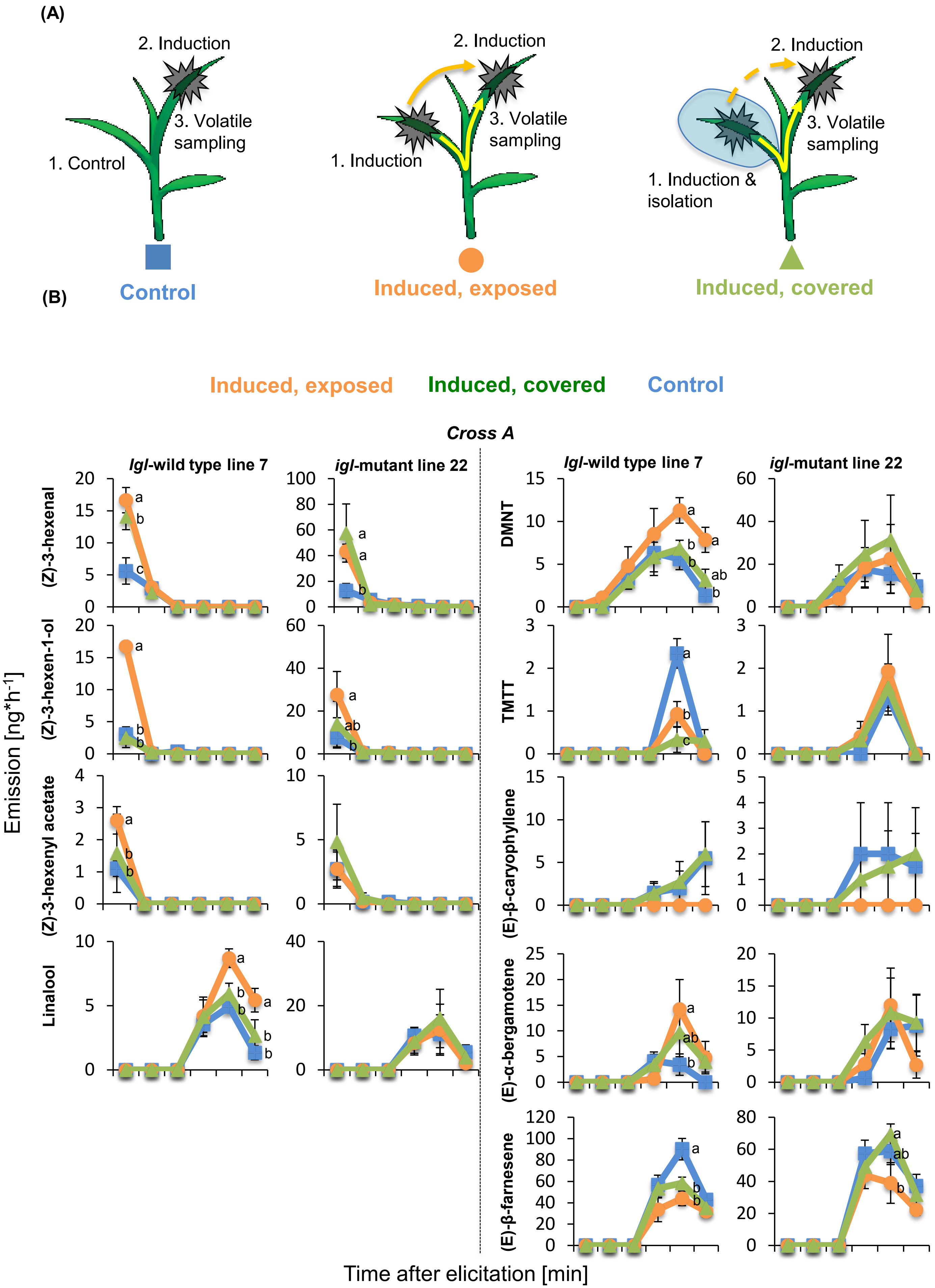

Supplementary Fig. 5: Individual HIPVs of experiment shown in figure 4. For experimental details, see figure 4.

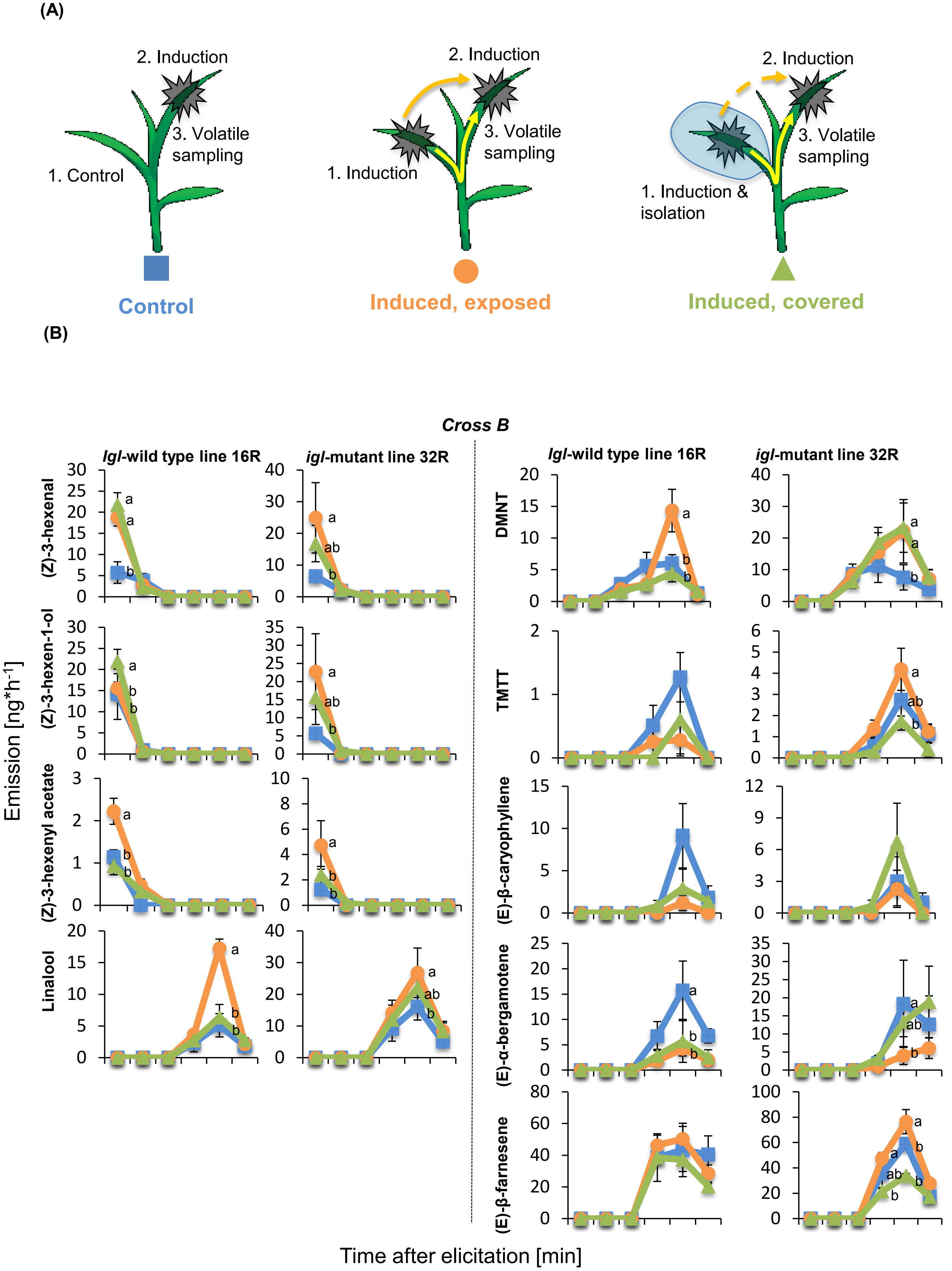

Supplementary Fig. 6: Individual HIPVs of experiment shown in figure 4. For experimental details, see figure 4.

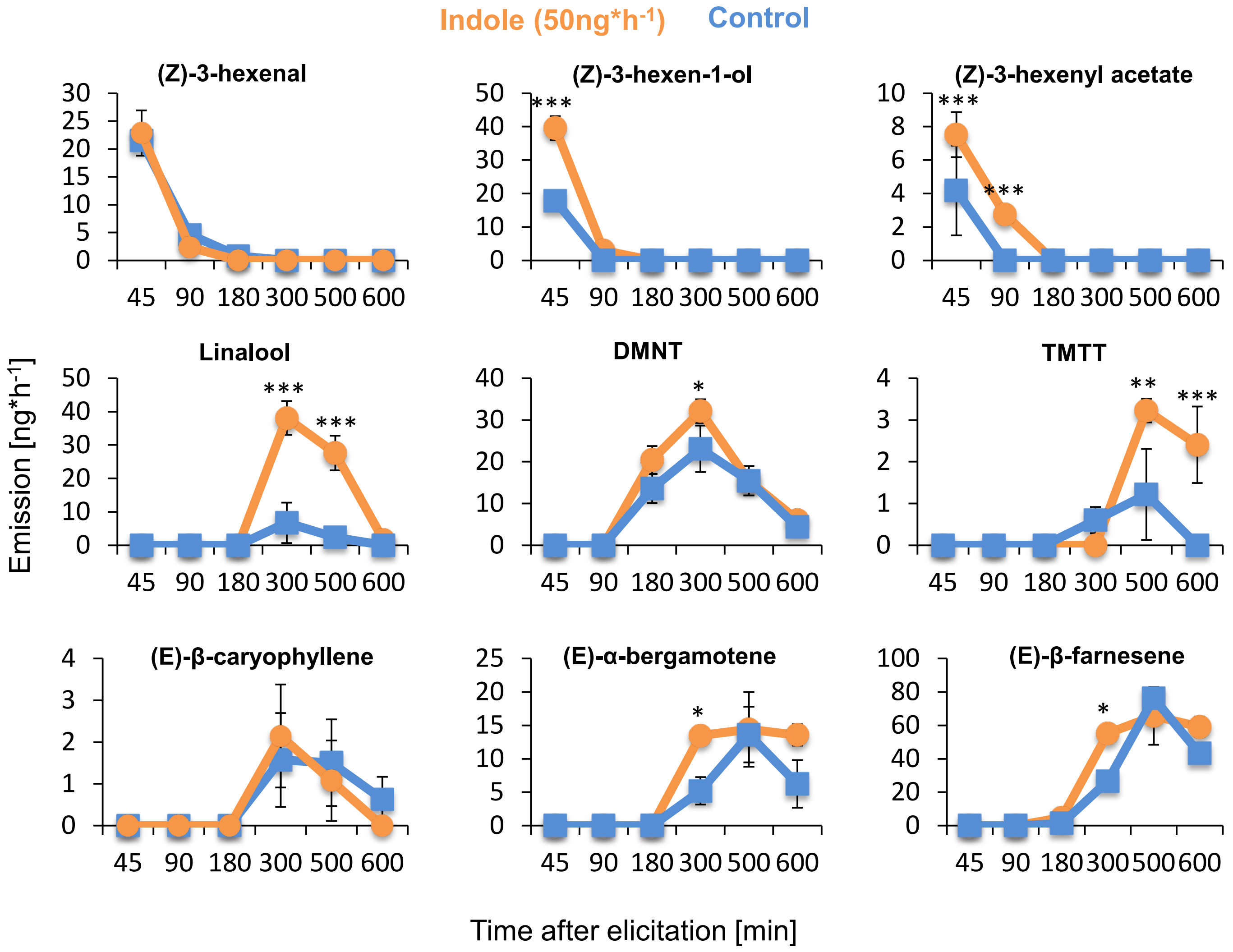

Supplementary Fig. 7: Individual HIPVs of indole-exposed mutant line 22. For experimental details, see figure 5.

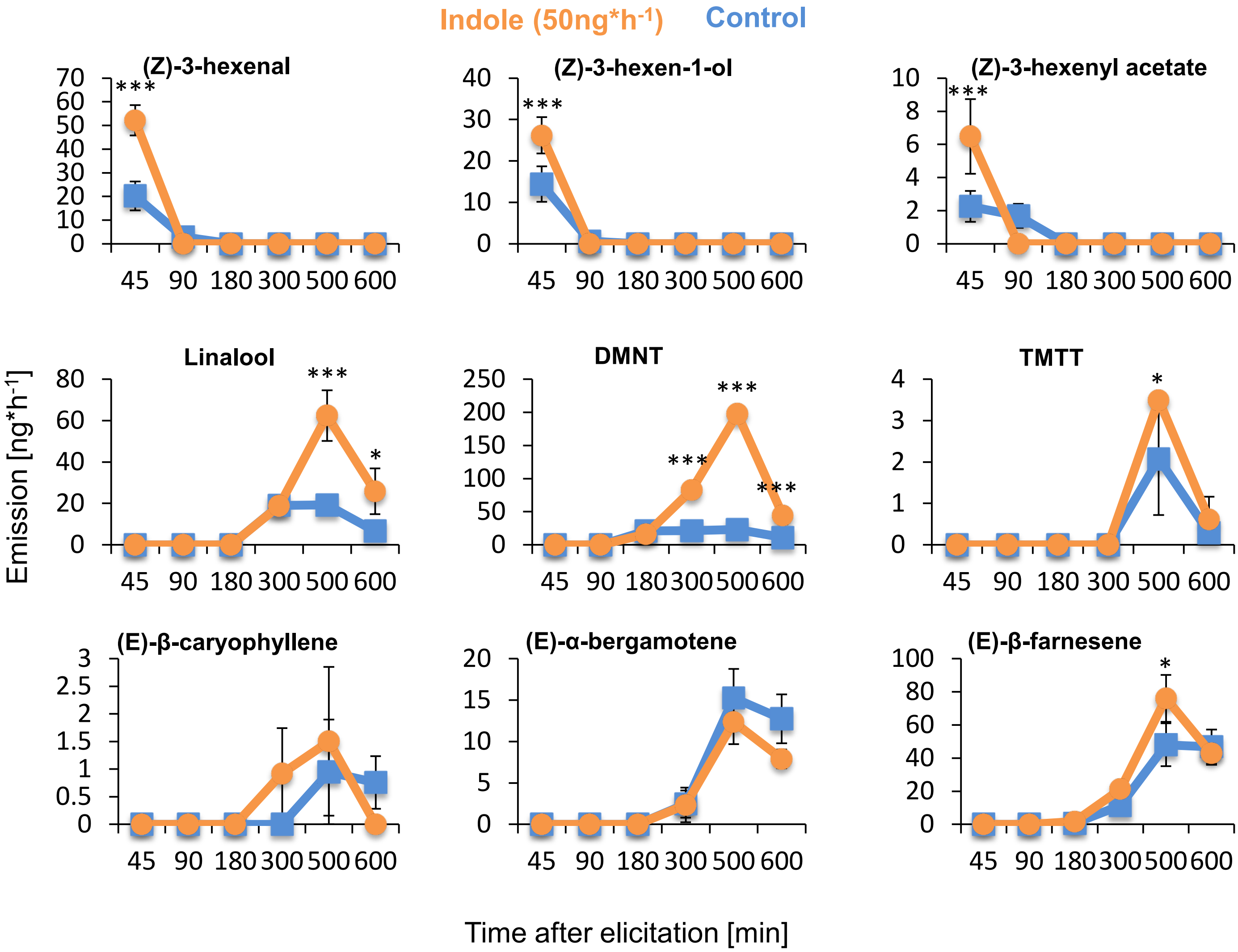

Supplementary Fig. 8: Individual HIPVs of indole-exposed mutant line 32R. For experimental details, see figure 5.

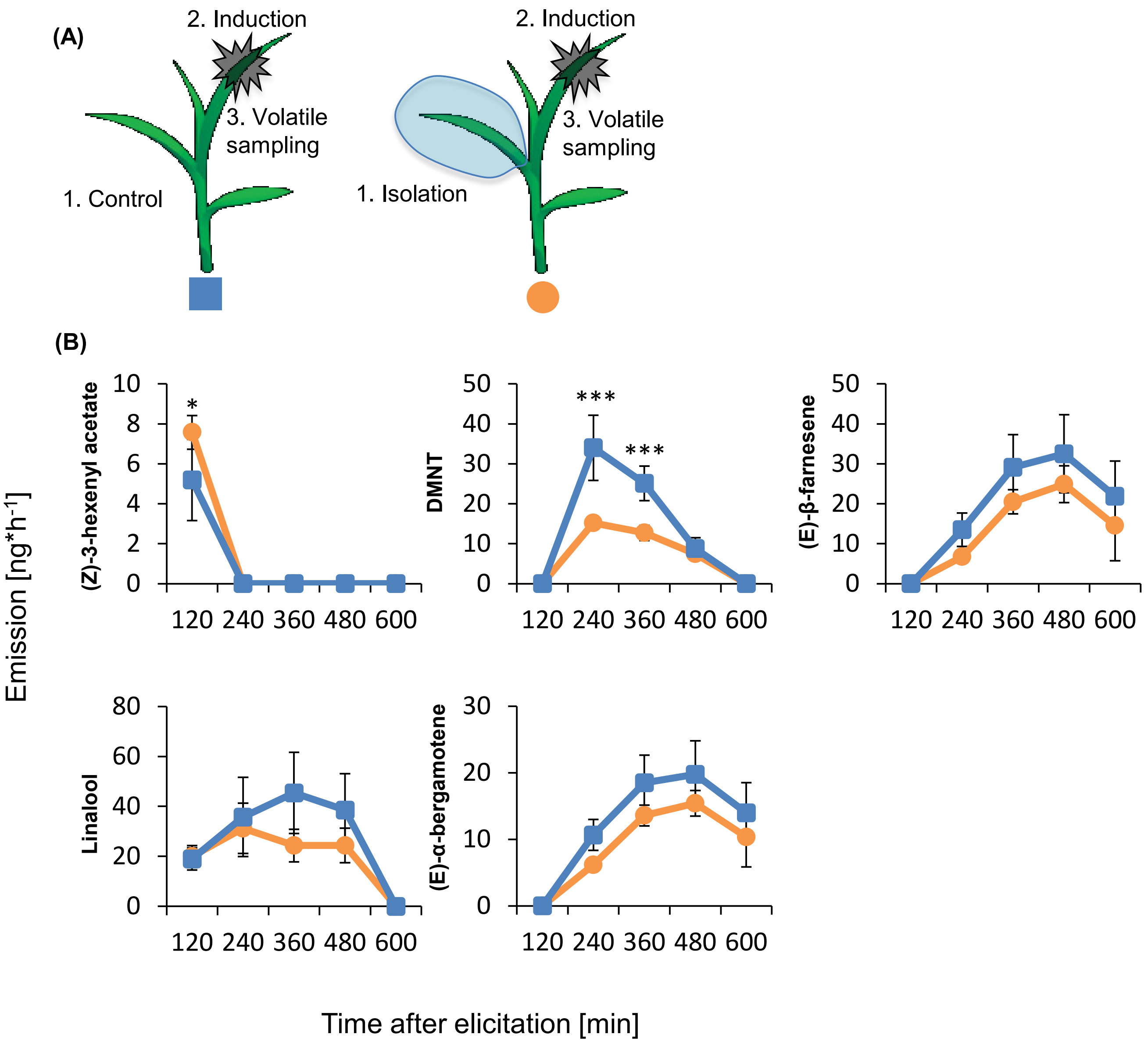

Supplementary Fig. 9: Effect of 12 h bagging on induced volatile emissions in the hybrid Delprim. The first true leaves of individual seedlings were wrapped in a Teflon bag for 12 h. The bags were then removed, and the second true leaf was induced by wounding and the application of *Spodoptera littoralis* regurgitate, followed by 2h volatile collections. Note that in this experiment, only major HIPVs could be detected. (*Z*)-3-hexenal, (*Z*)-3-hexen-1-ol, TMTT and (*E*)-β-caryophyllene were below quantification thresholds. Stars indicate significant differences between treatments (Holm Sidak post hoc tests, *:p<0.05, **:p<0.01, ***:p<0.001, n=3) . Error bars correspond to standard errors (±SE).

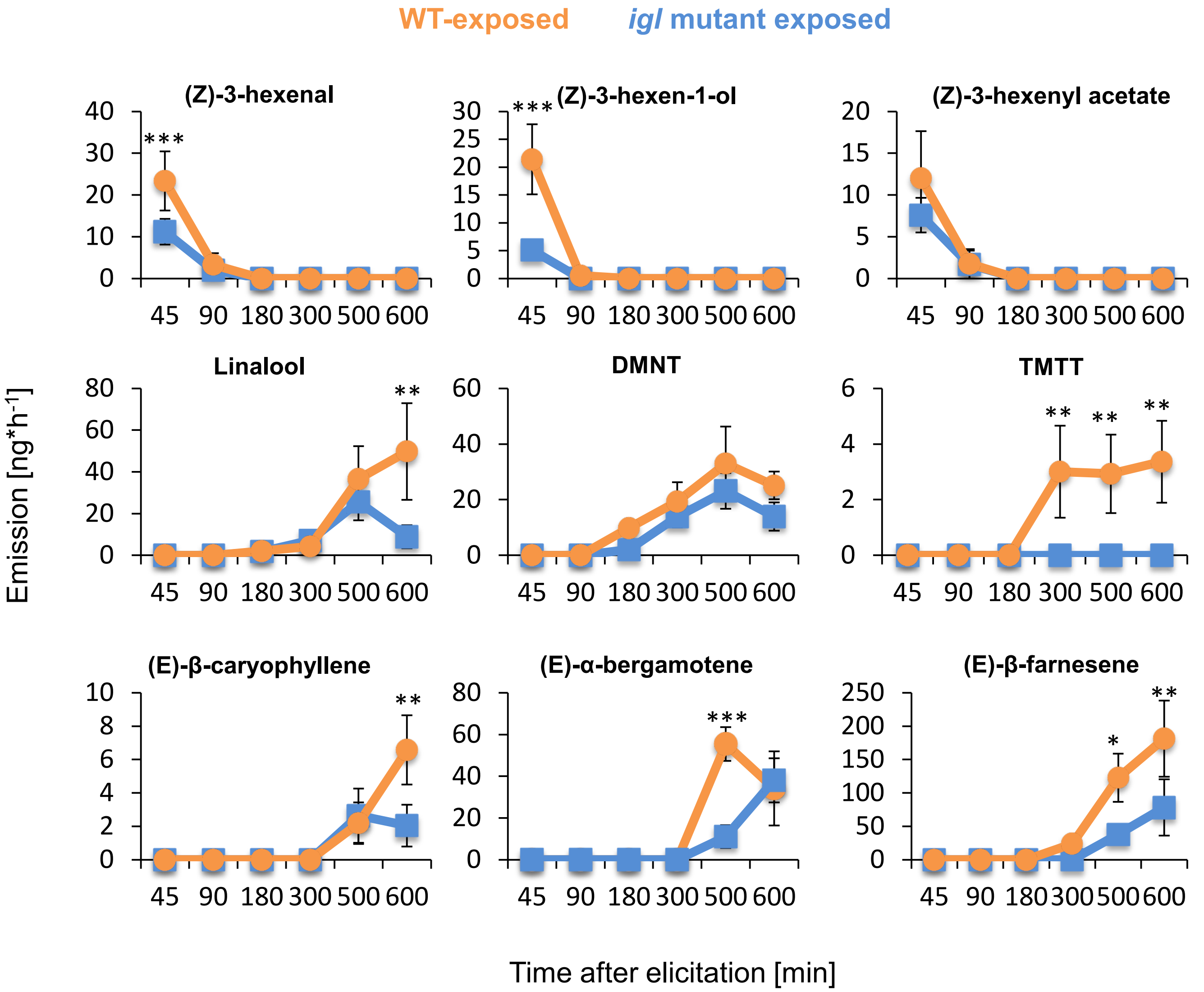

Supplementary Fig. 10: Individual HIPVs of experiment shown in figure 6 (Cross A). For experimental details, see figure 6.

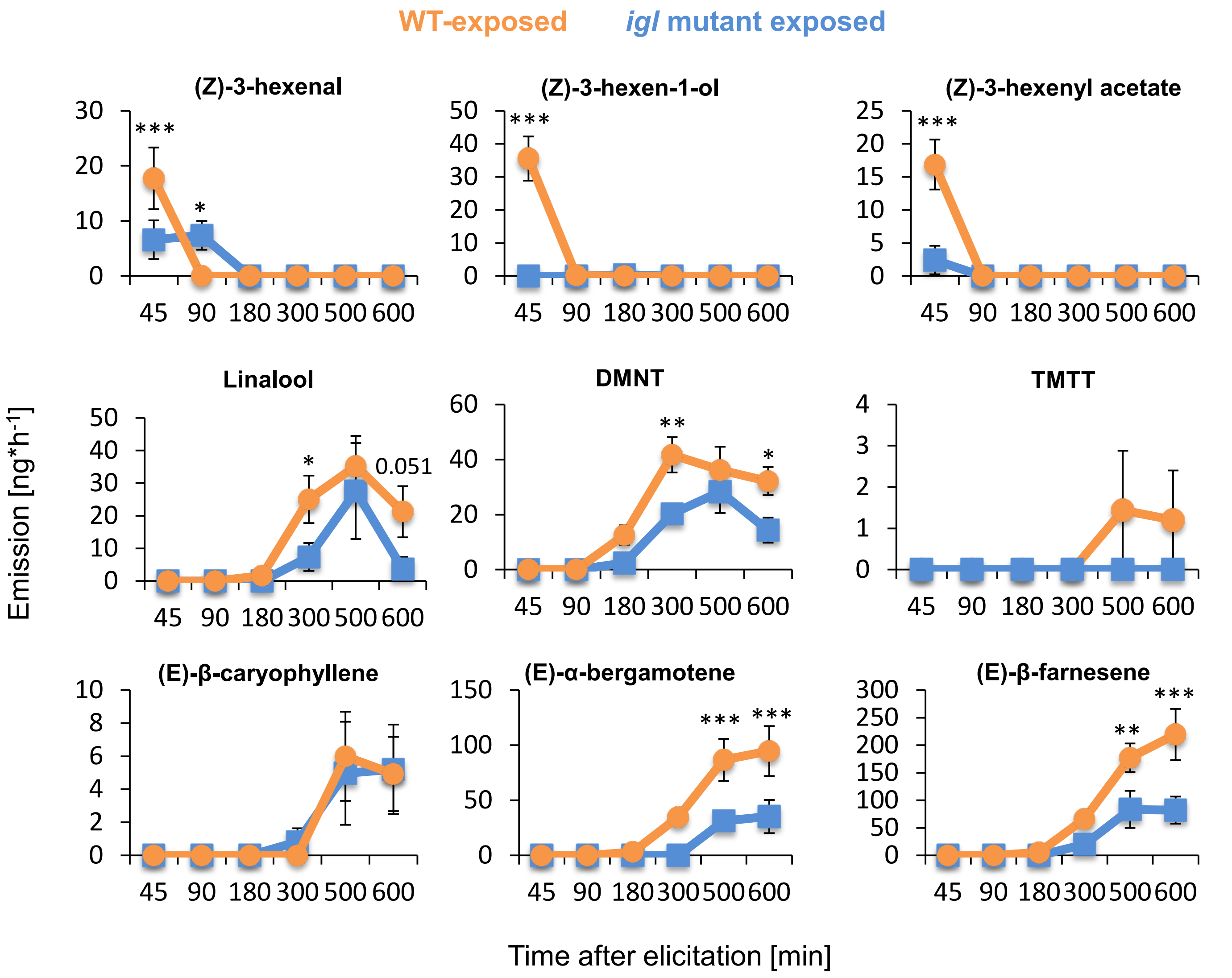

Supplementary Fig. 11: Individual HIPVs of experiment shown in figure 4 (Cross B). For experimental details, see figure 6.

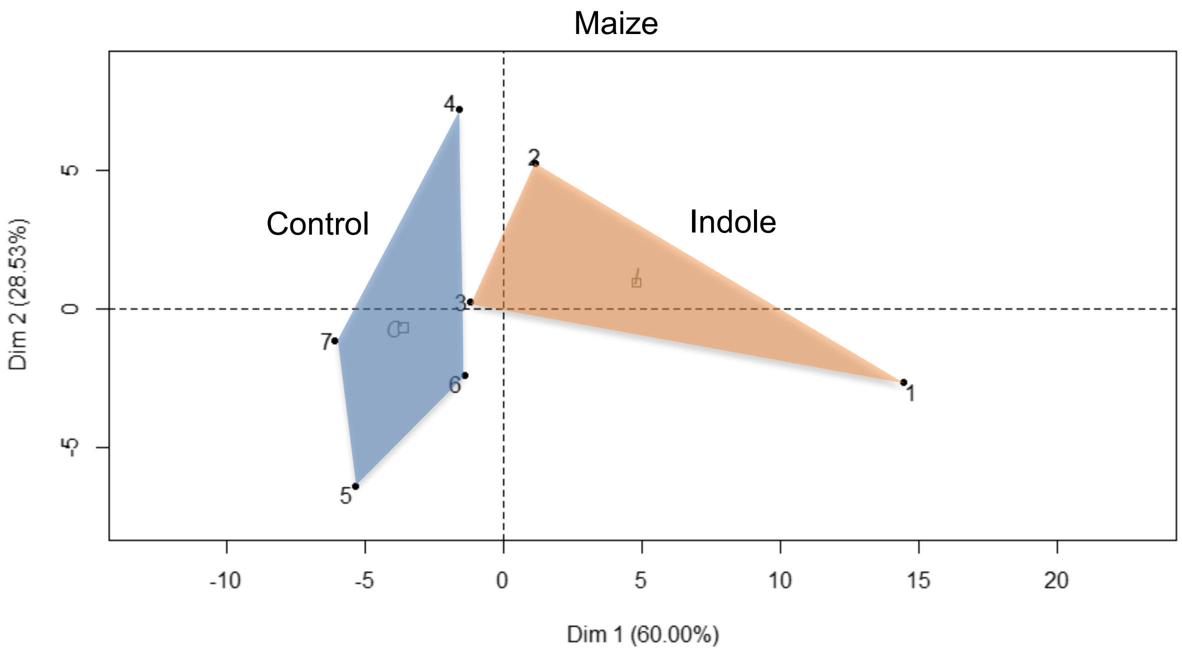

Supplementary Fig. 12: Principal component analysis (PCA) of indole-primed maize seedlings. Three-week-old maize plants were induced by wounding and S. littoralis regurgitate (n=3-4), and volatiles were collected from 180-630 minutes. Volatile features were detected using the GC/ single quad (centWave) algorithm in XCMS online, summed up over the sampling period and analysed using the PCA function of FactoMineR. Variables were scaled. The first two components of the PCA are depicted.

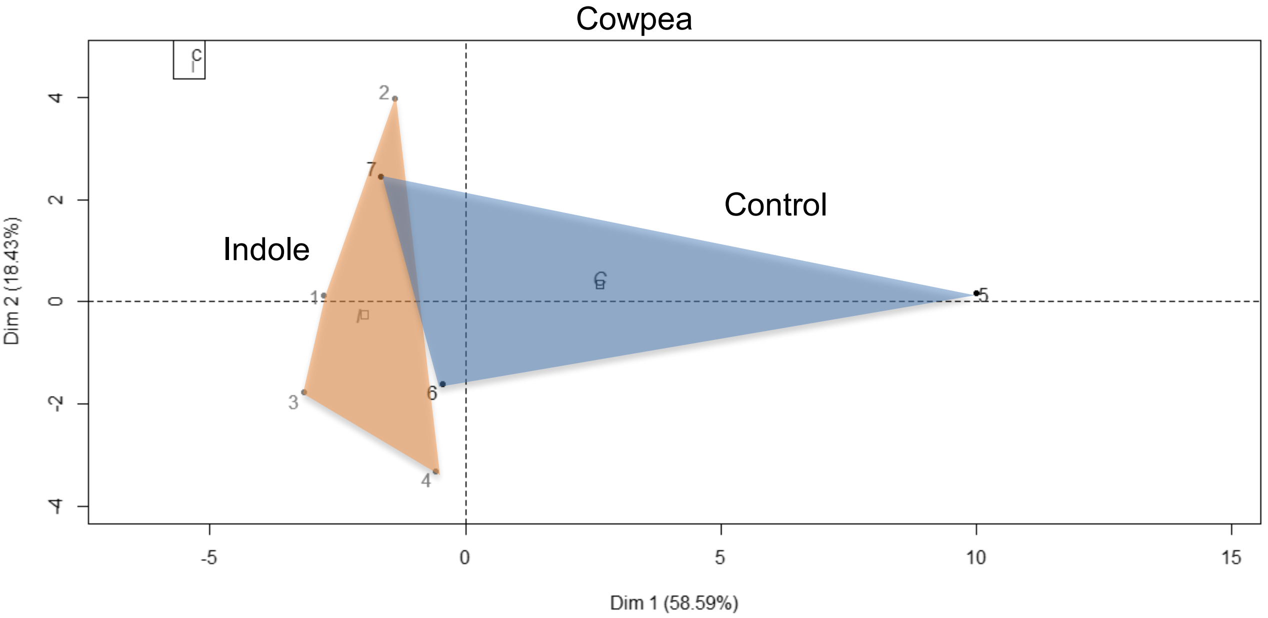

Supplementary Fig. 13: Principal component analysis (PCA) of indole-primed cowpea seedlings. Four-week-old cowpea plants were induced by wounding and *S. littoralis* regurgitate (n=3-4), and volatiles were collected from 180-630 minutes. Volatile features were detected using the GC/ single quad (centWave) algorithm in XCMS online, summed up over the sampling period and analysed using the PCA function of FactoMineR. Variables were scaled. The first two components of the PCA are depicted.

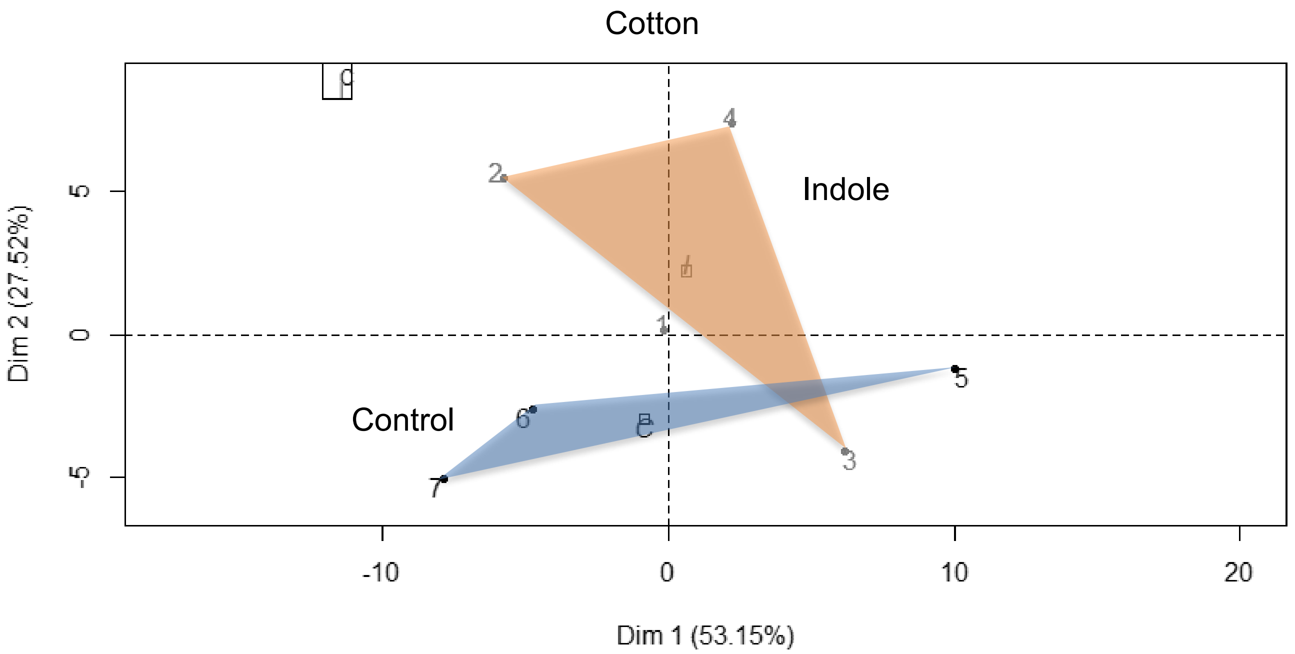

Supplementary Fig. 14: Principal component analysis (PCA) of indole-primed cotton seedlings. Four-week-old cotton plants were induced by wounding and *S. littoralis* regurgitate (n=3), and volatiles were collected from 180-630 minutes. Volatile features were detected using the GC/ single quad (centWave) algorithm in XCMS online, summed up over the sampling period and analysed using the PCA function of FactoMineR. Variables were scaled. The first two components of the PCA are depicted.
